# Photocatalytic Semiconductor–Metal Hybrid Nanoparticles: Single-Atom Catalyst Regime Surpasses Metal Tips

**DOI:** 10.1021/acsnano.4c13603

**Published:** 2025-01-06

**Authors:** Shira Gigi, Tal Cohen, Diego Florio, Adar Levi, David Stone, Ofer Katoa, Junying Li, Jing Liu, Sergei Remennik, Franco V. A. Camargo, Giulio Cerullo, Anatoly I. Frenkel, Uri Banin

**Affiliations:** † Institute of Chemistry, The Hebrew University of Jerusalem, Jerusalem 9190401, Israel; ‡ The Center for Nanoscience and Nanotechnology, The Hebrew University of Jerusalem, Jerusalem 9190401, Israel; § Dipartimento di Fisica, Politecnico di Milano, Milano 20133, Italy; ∥ Istituto di Fotonica e Nanotecnologie, Consiglio Nazionale delle Ricerche, Milano 20133, Italy; ⊥ Department of Materials Science and Chemical Engineering, Stony Brook University, Stony Brook, New York 11794, United States; # Department of Mathematics and Physics, Manhattan University, Riverdale, New York 10471, United States; ∇ Chemistry Division, Brookhaven National Laboratory, Upton, New York 11973, United States

**Keywords:** nanoparticles, photocatalysis, semiconductor−metal hybrids, single-atom catalyst, water splitting

## Abstract

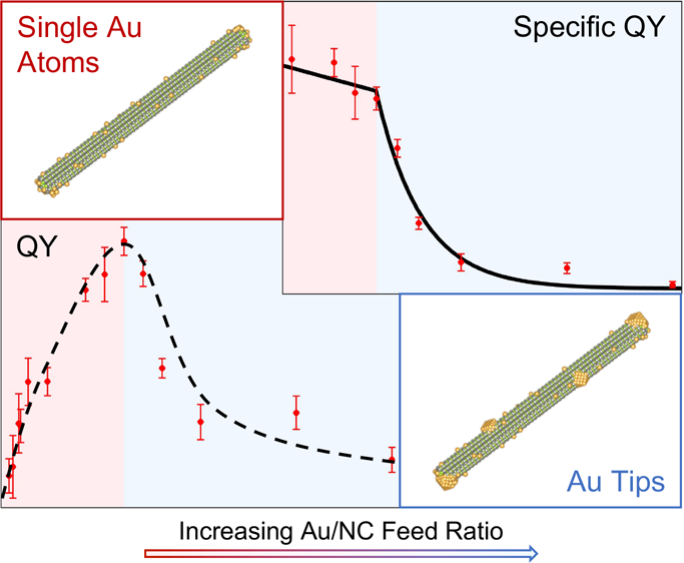

Semiconductor–metal hybrid nanoparticles (HNPs) are promising materials for photocatalytic applications, such as water splitting for green hydrogen generation. While most studies have focused on Cd containing HNPs, the realization of actual applications will require environmentally compatible systems. Using heavy-metal free ZnSe-Au HNPs as a model, we investigate the dependence of their functionality and efficiency on the cocatalyst metal domain characteristics ranging from the single-atom catalyst (SAC) regime to metal-tipped systems. The SAC regime was achieved via the deposition of individual atomic cocatalysts on the semiconductor nanocrystals in solution. Utilizing a combination of electron microscopy, X-ray absorption spectroscopy, and X-ray photoelectron spectroscopy, we established the presence of single Au atoms on the ZnSe nanorod surface. Upon increased Au concentration, this transitions to metal tip growth. Photocatalytic hydrogen generation measurements reveal a strong dependence on the cocatalyst loading with a sharp response maximum in the SAC regime. Ultrafast dynamics studies show similar electron decay kinetics for the pristine ZnSe nanorods and the ZnSe-Au HNPs in either SAC or tipped systems. This indicates that electron transfer is not the rate-limiting step for the photocatalytic process. Combined with the structural-chemical characterization, we conclude that the enhanced photocatalytic activity is due to the higher reactivity of the single-atom sites. This holistic view establishes the significance of SAC-HNPs, setting the stage for designing efficient and sustainable heavy-metal-free photocatalyst nanoparticles for numerous applications.

## Introduction

Semiconductor–metal hybrid nanoparticles (HNPs) manifest synergetic properties, in particular, light-induced charge separation upon light absorption by the semiconductor.^1−8^ This, accompanied by the enhanced catalytic functionality of the metal sites, leads to improved charge extraction, thus promoting HNPs as promising photocatalysts for various applications,^9−11^ including green hydrogen generation via water splitting,^12−17^ CO_2_ reduction,^18,19^ and photoinitiation of radical polymerization.^20,21^ Since the first demonstration of colloidal HNPs based on Au-tipped CdSe nanorods (NRs), a variety of such systems have been developed, achieving control over the size, position, and morphology of both the semiconductor and the metal components. Recently, the ongoing effort to achieve efficient green photocatalysts has encouraged the development of heavy-metal-free HNPs.^22−27^

The size and number of metal domains were found to strongly affect the photocatalytic efficiency of the HNPs. An optimal metal tip size for hydrogen generation by water splitting was found for CdS-Au HNPs and attributed to an interplay between higher charge separation efficiency and the loss of overpotential for larger tips, thus balancing the rates of these two consecutive steps in the water reduction reaction.^28^ In the case of Ni-tipped CdS NRs, the discovered optimal domain size for hydrogen generation was attributed to the development of a Schottky barrier at the semiconductor–metal interface.^29^ CdS-Pd also showed the nonmonotonic dependence of the photocatalytic efficiency on the metal tip size,^30^ while in CdS-Pt a monotonic increase was detected, up to the studied size of 3 nm.^31^

In terms of the number of metal domains, the study on CdS NRs showed improved efficiency for a single domain over two or many domains, assigned to the beneficial concentration of charges for the two-electron reduction reaction.^32,33^ Later studies on the optimal number of metal clusters in ZnSe-Pt HNPs revealed a similar trend.^27^

In this work we address an interesting regime for the HNPs in the “single-atom” catalyst (SAC) limit, in which isolated monometallic sites of the cocatalytic metal are deposited on the surface of the semiconductor nanocrystals (NCs).^34^ The SAC regime was studied in numerous systems, including oxides-supported noble metals, such as Pt, Au, and Pd on CeO_2_, Al_2_O_3_, and FeO_*x*_.^34−37^ Later on, the SAC concept was extended to include additional transition metals (Fe, Co, Zn, etc.) supported on different metals, carbon-based materials, and metal–organic frameworks.^37−44^

The ionic nature of the catalytic sites and the lack of metal–metal bonds in the SAC regime give rise to fascinating geometric and electronic properties. For thermocatalysis in various systems, the SACs exhibit exceptionally high performance, compared with their metallic nanoparticle equivalents, in terms of catalytic activity, atom utilization efficiency, chemoselectivity, and stability.^36,37,40,45−49^ Furthermore, the unique structure–activity correlation of the SAC regime has recently driven the utilization of SAC-based systems for electro- and photocatalysis, which require a more complex balance of catalyst properties, including conductivity, light absorption, structural aspects, and electro- and photostability.^37−39,42,47,50−54^

While the SAC regime has been studied in numerous catalytic materials,^34−43^ only a handful of works addressed this for photocatalytic Cd-based semiconductor NCs.^55−62^ Atomically dispersed Co-P_3_ species on CdS NRs showed enhanced efficiency for dehydrogenation of formic acid to hydrogen, due to their electron-rich feature facilitating charge separation.^55^ In the case of atomically dispersed Ni in Cd_1–*x*_Zn_*x*_S quantum dots, the optimal loading, with a high density of monovalent Ni on the (111) facet, yielded over 10-fold enhancement in hydrogen generation.^56^ In Pt-CdSe nanoplatelets, surface-adsorbed Pt exhibited enhanced hydrogen generation and improved stability, whereas interior Pt atoms substituting Cd showed substandard performance due to strained coordination geometry.^58^ These studies showcase the potential and merit of the SAC regime on semiconductor NCs as supports and sensitizers. However, the SAC regime in heavy-metal-free semiconductor NCs, which are important for environmental compatibility, has not been reported yet. Furthermore, studying the transition between the SAC regime and the metal-tipped systems has not yet been addressed, which is important for further understanding of the mechanisms contributing to photocatalytic functionality in HNPs.

Herein, we report on the synthesis and characterization of heavy-metal-free ZnSe-Au HNPs in the SAC regime and compare their functionality with the metal-tip regime. The choice of Au stems from the ease of synthetic control of its growth on the ZnSe nanorods. The presence of single Au atom sites on the surface is proven by a combination of high-resolution scanning transmission electron microscopy (HR-STEM), X-ray absorption spectroscopy (XAS), and X-ray photoelectron spectroscopy (XPS). The efficiency of photocatalytic hydrogen generation shows a strong dependence on the cocatalyst loading, with a sharp maximum in the SAC regime, decreasing toward the metal-tip limit. Ultrafast transient absorption (TA) studies are performed to investigate the light-induced charge transfer.^2,13,14,29,63^ The similar charge carrier dynamics in both the SAC and metal-tip regimes reveal the dominance of trapping processes over the metal site characteristics upon near-band-edge excitation. Combined with the structural-chemical studies, this indicates that the photocatalytic functionality is not governed by the electron transfer rate but rather determined by the chemical reactivity of the metal site. Thus, by characterizing the structural, chemical, and light-induced carrier dynamics of the HNPs, we obtain a holistic view of their photocatalytic performance. The study broadens the path for designing efficient heavy-metal-free HNP-based photocatalysts in the SAC limit.

## Results and Discussion

### Synthesis of ZnSe-Au Hybrid Nanoparticles

**Figure 1:**
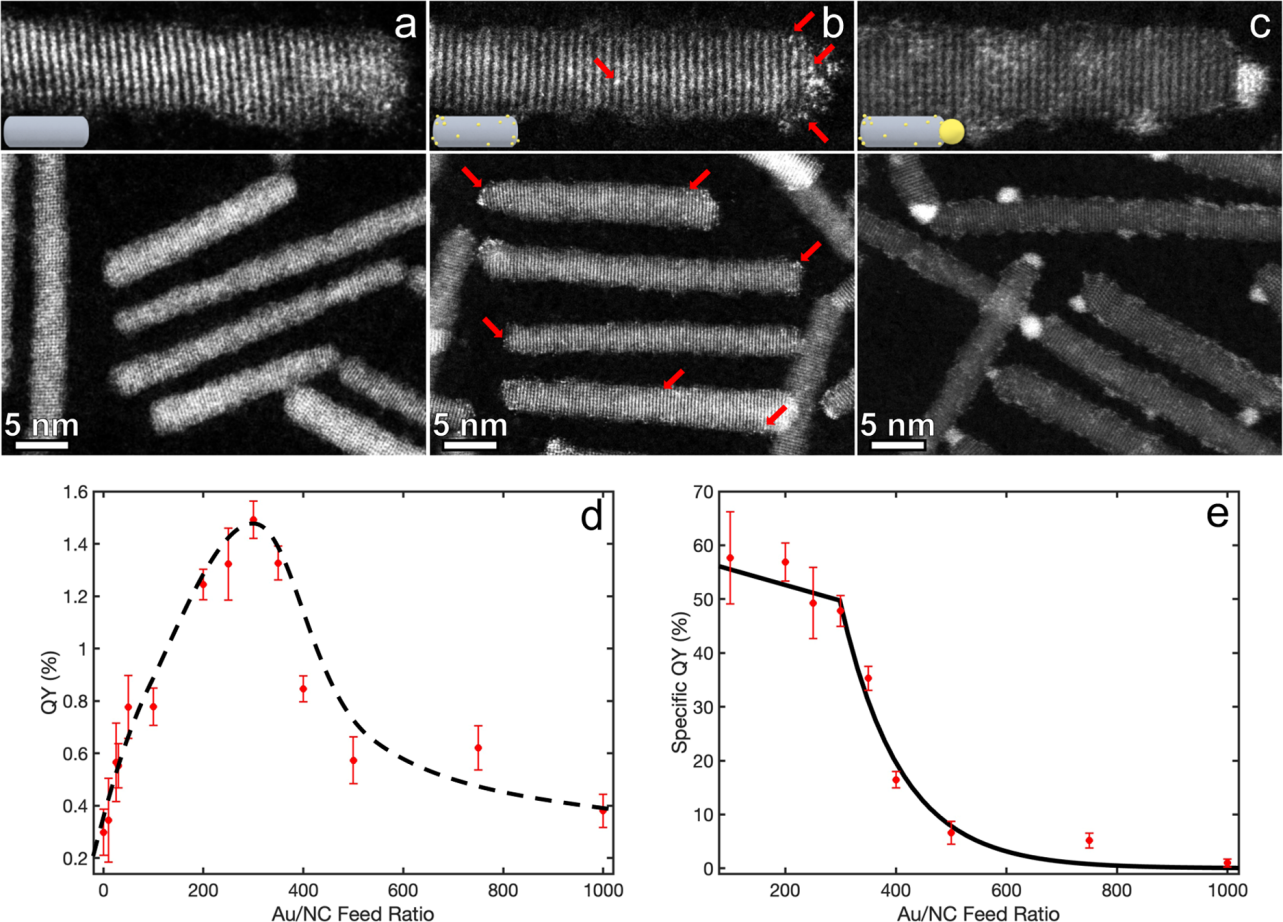
Synthesis and activity. (a–c) HAADF-STEM images of: (a) ZnSe NRs, (b) ZnSe-Au HNPs in the SAC regime (100 Au/NC). Atomic Au sites appear as bright spots on the NRs surface (examples marked by red arrows). (c) ZnSe-Au HNPs in the metal-tip regime (1000 Au/NC). Insets show illustrations of the different regimes. (d) Photocatalytic QY of ZnSe-Au HNPs for hydrogen generation versus the Au/NC feed ratio. The optimal QY was measured for the 300 Au/NC ratio, corresponding to the SAC limit. The HNPs manifest a strong dependence of the QY on the Au/NC ratio with a sharp peak in the SAC limit, demonstrating the high efficiency of the SAC regime. (e) Specific QY of the HNPs calculated by normalizing the QY to the Au loading. The SAC regime shows a linear dependence in the feed ratio with a minor decrease in the specific QY for increasing loadings. Above 300 feed ratio, upon the formation of metal tips, the specific QY decreases significantly for increasing Au loadings.

ZnSe NRs were synthesized via a heat-up method using a previously reported procedure with minor modifications (see Experimental Section).^27^ Briefly, Zn acetate dihydrate, Se powder, and 1-dodecanethiol were dissolved in oleylamine and heated to 110 °C to form ZnSe clusters, then heated to 220 °C to obtain ZnSe nanowires, and finally heated to 270 °C to obtain ZnSe NRs. Figure 1a shows HR-STEM images of the pristine ZnSe NRs, indicating that they are 30 ± 9 nm in length and 2.6 ± 0.5 nm in diameter. The absorbance spectrum of the NRs shows an excitonic peak at 372 nm, shifted from the ZnSe bulk band gap (∼460 nm) by the quantum confinement effect dictated by the small diameter (Figure S1). The as-synthesized ZnSe NRs were capped with oleylamine surface ligands, providing colloidal stability in organic media.

To address the main research question concerning the effect of the metal domain characteristics on photocatalysis, Au was chosen due to the relative ease of the synthetic control over its deposition and growth reaction. We synthesized ZnSe-Au HNPs by mixing the NRs with AuCl_3_ as the Au precursor and oleylamine as a reducing agent under an ambient atmosphere in the dark (see the Experimental Section). To synthesize a series of HNPs with varying Au loadings, we changed the Au/NC feed ratio, namely, the molar ratio between the Au salt and the NRs in the metal deposition reaction while keeping the total concentration and the oleylamine/AuCl_3_ ratio constant for all HNPs.

The size and distribution of Au in the final products were characterized via HR-STEM equipped with a high-angle annular dark-field (HAADF) detector (Figure 1). Due to its high sensitivity to the atomic number (*Z*), HAADF-STEM detects even small amounts of Au (down to a single atom) on the lower-*Z* ZnSe lattice, appearing as brighter regions in the STEM images. HR-STEM images with a wider field of view are available in the Supporting Information (Figure S2).

Figure 1b shows the HAADF-STEM images of HNPs synthesized with a feed ratio of 100. Numerous bright spots on the NR body are assigned to single Au atoms (examples indicated by arrows). The dispersion of the Au atoms changes in different regions of the NRs, with a uniform dispersion along the rod body and higher accumulation areas around the apexes. This is attributed to the higher reactivity of the apex regions. The curvature of the apexes leads to a lower density of ligands and a higher concentration of dangling bonds, facilitating the attachment of Au to the surface of the NRs. In related CdS systems, the Se-rich facet further leads to higher reactivity of that apex region.^1,7^

Going to higher Au loadings, Figure 1c shows HAADF-STEM images of HNPs synthesized with a feed ratio of 1000 Au/NC. Here, crystalline Au tips are formed mainly on the apexes. Additionally, for this high Au loading the dispersion of the Au atoms along the rods' body is not uniform, they accumulate in different regions on the NRs and even form some Au clusters along the rods' body.

These two Au/NC feed ratios represent two different limits—the SAC regime and the regime of metal tips. For low Au/NC feed ratios (<400) the SAC regime dominates where higher feed ratios result in larger numbers of isolated Au atoms on the ZnSe NRs. In the intermediate region, larger amounts of Au atoms accumulate near the apexes, leading also to the formation of small Au clusters on some NRs.

For the higher feed ratios, we observed Au clusters on the majority of the NRs and the growth of larger Au tips on the NRs, while the dispersion of single Au atoms along the rod body remains mostly uniform. For this metal-tip regime, increasing Au loadings leads to gradual growth of the Au domains on the apexes, forming crystalline Au tips.

In order to examine the stability of the obtained SAC-HNPs, we characterized their structural changes upon exposure to air and heat. SAC-HNPs with a feed ratio of 150 Au/NC were dispersed in toluene and heated to 80 °C under an ambient atmosphere. Then, we characterized the HNPs via HR-STEM and UV–vis absorption (Figure S3). The stability experiments indicated no structural or spectral change during the first 6 h, as the SACs retain their homogeneous dispersion on the ZnSe surface. Moreover, the sintering of Au atoms occurred only after 24 h of exposure to heat and air, forming subnanometric Au clusters. Nonetheless, most Au atoms remained isolated and homogeneously dispersed on the NR surface, and no spectral change was observed. Eventually, after 56 h of heating, the Au atoms formed small tips, followed by redshift and spectral broadening. However, by that stage, the HNPs had started to disassemble, as both Au and ZnSe clusters were detached from the surface due to prolonged heating.

### Photocatalytic Activity

We studied the photocatalytic activity of ZnSe NRs and ZnSe-Au HNPs, focusing on the formation of green hydrogen via water splitting. We synthesized ZnSe-Au HNPs with varying Au loadings, ranging from SAC to the metal-tip regime. The Au/NC feed ratio in the Au deposition reaction was chosen carefully to span each regime and to observe the transition between the SAC and metal tips in a precise manner. To conduct photocatalytic measurements in aqueous media, the hydrophobic oleylamine surface ligands were exchanged and the HNPs were capped with polyethylenimine (PEI, see Experimental Section).^64^ This branched hydrophilic polymer contains primary, secondary, and tertiary amine groups, binding to the NR surface and providing colloidal stability in water. Photocatalytic hydrogen generation was then measured under 365 nm illumination using a gas chromatograph (GC) equipped with a thermal conductivity detector (Figure S4). Ascorbic acid was used as a hole scavenger to facilitate the water reduction reaction (see Experimental Section).

To evaluate the photocatalytic efficiency of the HNPs, the photocatalytic quantum yield (QY) for hydrogen generation was calculated from the data (see the Supporting Information, Photocatalytic Activity). Figure 1d presents the calculated QY for ZnSe-Au HNPs versus the Au/NC feed ratio. The HNPs exhibit a clear nonmonotonic dependence of the QY on the Au loading, with a sharp response peak for a 300 Au/NC feed ratio. For the SAC regime, with up to a 300 Au/NC feed ratio, the deposition of atomic Au sites on the NR body dramatically improves the efficiency. The optimal Au loading (300 feed ratio) reaches a maximal QY of 1.5%, whereas pristine ZnSe shows poor activity with less than 0.3% QY. For higher Au loadings, corresponding to the formation of Au tips, the efficiency decreases with increasing Au loadings. A significant drop of more than 30% is observed when increasing the feed ratio from 350 to 400 Au/NC. Above the 400 feed ratio, the efficiency moderately decreases, eventually reaching less than 0.4% QY for the 1000 feed ratio, which is comparable to the performance of pristine ZnSe.

To further analyze the transition between SAC and tips, we calculated the specific QY of the Au atomic sites by subtracting the QY of pristine ZnSe and dividing by the Au loading (see the Supporting Information, Photocatalytic Activity). Figure 1e presents the specific QY (per Au atom) of the measured HNPs. For the SAC regime (up to 300 feed ratio), the specific QY is nearly constant within error, showing only a minor linear decrease for increasing Au loading. This indicates a similar constant catalytic contribution of each single Au atom deposited on the ZnSe surface, a clear signature characteristic of the SAC regime.^35^ The small decrease in specific QY is attributed to the partial accumulation of metal atoms, which exceeds the single-atom regime. For Au/NC feed ratios larger than 300 we observe a significant decrease in the specific QY for increasing Au loadings. The strong decrease in specific activity eventually results in a negligible specific QY for the 1000 feed ratio, which explains the comparable QYs observed for the 1000 feed ratio and pristine ZnSe.

In order to examine the stability of the HNPs during the photocatalytic reaction, their structure was further characterized via HAADF-STEM after the reaction (Figures S5 and S6). The STEM analysis shows that for the metal-tip regime (1000 Au/NC) the tips are maintained, and no change is seen. For the highest SACs loading (300 Au/NC), there is a partial aggregation of Au, forming small clusters on some of the NRs, which is also the case before the photocatalytic reaction. Notably though, for 100 Au/NC feed ratio, the STEM images indicate no clusters but rather a uniform dispersion of single Au atoms on the NRs surface, confirming the stability of the SACs. Essentially, therefore, the HNPs in both regimes, SACs and tips, retain their original Au configuration (see further discussion in the Supporting Information).

The nonmonotonic dependence of the photocatalytic activity on the Au/NC feed ratio, with a sharp response around the transition from the SAC to the metal-tip regime, indicates an intricate mechanism involving not only the Au loading but also the nature of the Au site.

### Structural-Chemical Characterization

The interesting dependence of the photocatalytic efficiency on the Au deposition regime calls for further scrutiny of the nature of the Au sites in each case. To this end, we utilized X-ray absorption fine structure (XAFS) measurements comparing HNPs in the SAC and metal-tip regime. XAFS measurements were used to validate the new SAC regime and provide further information about the coordination environment and oxidation states of Au in both regimes.

The XAFS data were collected at the NSLS-II synchrotron at Brookhaven National Laboratory, beamline 7-BM (QAS). The data were collected in fluorescence mode for the Au L_3_-edge and in transmission mode for Zn and Se K-edges. The details on the data analysis are given in the Supporting Information. For the comparison between SACs and tips, we chose representative samples with a Au/NC feed ratio of 80 and 1000, respectively, which were dropped from solution onto Kapton film (see Experimental Section).

**Figure 2:**
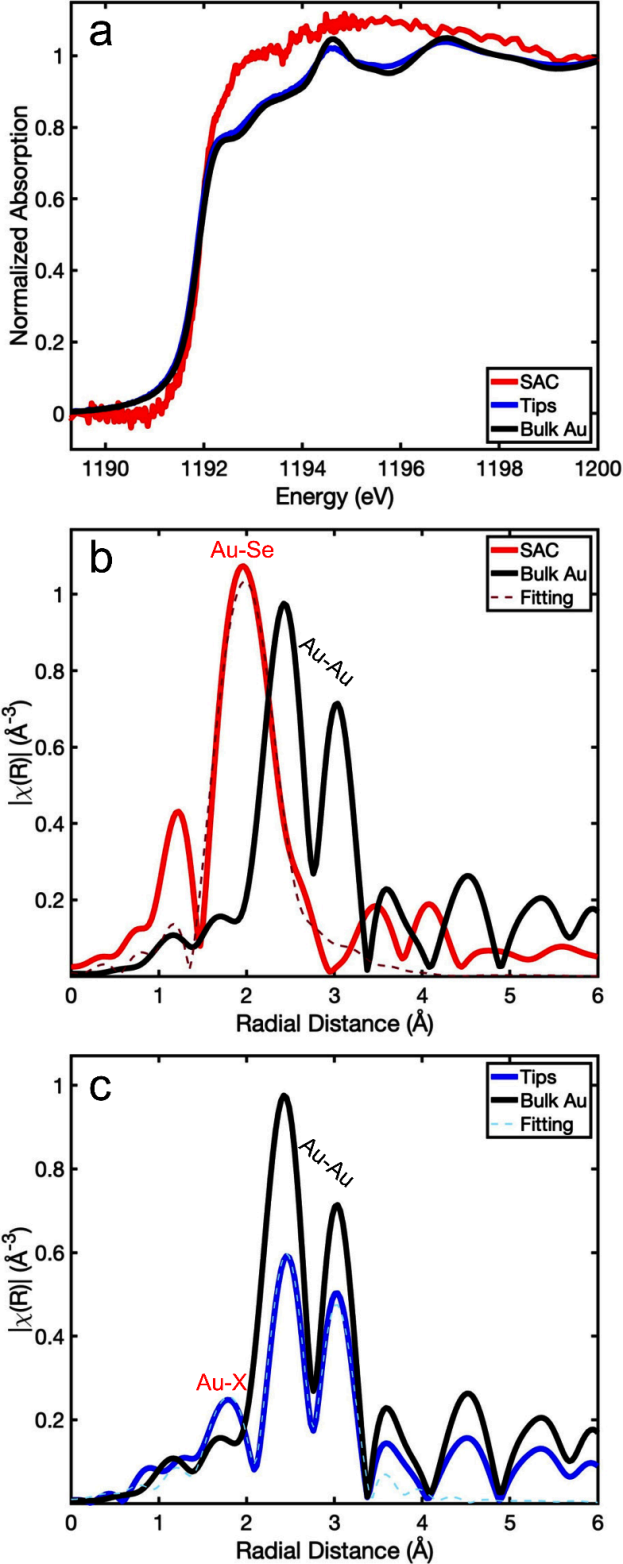
XAFS characterization. (a) Normalized Au L_3_-edge XANES spectra of ZnSe-Au HNPs in the SAC (red) and tips (blue) regime, compared to that of a reference Au foil sample corresponding to bulk Au (black). (b, c) *R*-space spectra of ZnSe-Au HNPs in the SAC and tips regime, respectively. The SAC-HNPs spectrum differs significantly from that of the bulk Au, showing different peak positions assigned to the presence of atomic Au sites. In contrast, the tipped NRs spectrum shows the same peak positions as bulk Au and differs mainly in peak intensities, corresponding to the lower coordination number typical for the nanoscale dimensions of the Au clusters. The fitted models (dashed lines) show exclusively Au–Se bonds for the SAC-HNPs, whereas the metal-tip regime comprises coexisting Au–Se and Au–Au bonds. The XAFS processing and fitting details are elaborated in the Supporting Information (Figure S7b and Table S1).

Figure 2a shows the X-ray absorption near edge structure (XANES) spectra corresponding to the Au L_3_-edge of SAC-HNPs (red) and Au-tipped NRs (blue). As a reference, we also measured a sample of Au foil corresponding to bulk Au (black). Evidently, the XANES spectrum of SAC-Au differs significantly from that of the Au foil. In contrast, the spectrum of the sample with Au tips exhibits features similar to those of bulk Au, indicating the presence of metallic Au surrounded by other Au atoms. This trend is also reflected in the extended XAFS (EXAFS) spectra in the *k*-space of the three samples presented in Figure S7a, where SAC-Au is distinguishable from the comparable spectra of Au tips and bulk Au.

To further study the oxidation states and coordination chemistry of Au in the different regimes, we compared the *r*-space (real-space) spectra of SAC-HNPs, Au-tipped NRs, and Au foil. The *r*-space spectra were obtained by the Fourier transform of the *k*^2^-weighted χ(*k*) in the range 2–10 Å^–1^. Figure 2b presents the Au L_3_-edge *r*-space spectra of ZnSe-Au HNPs in the SAC regime (red) and of the reference Au foil (black). The dashed line represents the fitted model for the SAC-HNPs. The *r*-space spectrum of the SAC-Au shows one dominant peak at a radial distance of 2 Å, significantly shifted from the two Au–Au peaks of bulk Au (2.4 and 3 Å). Quantitative analysis of the SAC-Au spectrum reveals a single contribution from the Au–Se scattering path and no evidence of Au–Au contributions (Supporting Information, Figure S7b and Table S1). This establishes the atomic dispersion of Au. Moreover, the average coordination number of Au is 2.9 ± 0.7, lower than what is expected for Au as a substitutional dopant in ZnSe (coordination number of 4).^65^ All in all, this provides further strong evidence for the deposition of single Au atoms on the surface of the NRs.

In contrast, the Au L_3_-edge *r*-space spectrum of the metal-tip regime shows a clear similarity to the Au foil, indicating a significant contribution arising from the Au–Au metallic bonds in the tips (Figure 2c). At a radial distance larger than 2 Å, the spectrum of Au-tipped ZnSe (blue) portrays similar features to those of bulk Au (black). The two main peaks at 2.5 and 3 Å are attributed to the first nearest neighboring Au–Au scattering paths, with the lower peak intensities indicating a lower coordination number compared to bulk Au, typical for small Au clusters with large fraction of surface atoms.

Noticeably, an additional peak (labeled Au-X) appears at 1.8 Å. Fitting the data by two contributions, Au–Se and Au–Au, as detailed in the Supporting Information, shows this feature to be an outcome of interference between the two paths. The result is the visible shift of the Au-X peak in Figure 2c to lower distances compared to the Au–Se peak in Figure 2b. Overall, this indicates the coexistence of two Au species: a metallic one, in which Au atoms are bonded to Au, and an ionic species, in which Au atoms are bonded to Se.^66^ Additional insight is obtained from quantitative analysis of the spectrum (dashed line in Figure 2c shows the fitted model for Au tips, as detailed in the Supporting Information (Figure S7b and Table S1).

In order to further interpret the XAFS data, several structural models were considered. Two limiting scenarios need to be considered: (1) a single population of Au clusters bound to the ZnSe NRs. Their size and structure dictate the ratio of internal Au–Au bonds versus interfacial Au–Se contributions. (2) Two populations, one of Au clusters (considered as contributing Au–Au interactions) and a second population of single Au atoms on the ZnSe surface, corresponding to Au–Se bonds. A detailed discussion of the two limits and the related models is described in the Supporting Information.

At this point, it is important to consider also the HAADF-STEM data (Figures 1c and S2d), directly showing the coexistence of several Au phases, including isolated atoms and clusters of different sizes. The correlation of the ensemble-averaged XAFS data and the local STEM imaging thus establishes the second scenario above of coexisting clusters and “single atoms”.^67^

**Figure 3:**
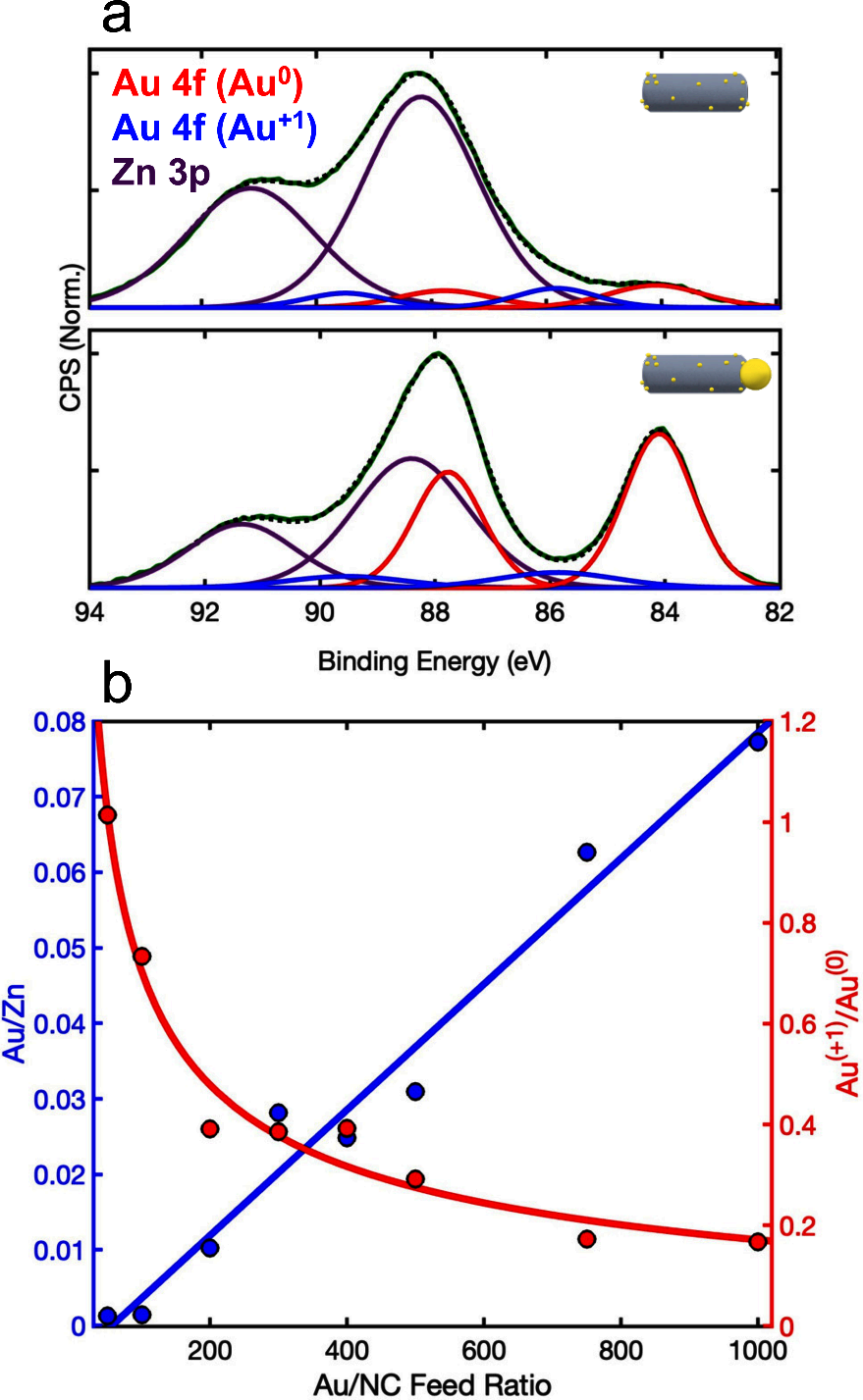
XPS analysis. (a) Normalized XPS spectra of ZnSe-Au HNPs in the SAC (top) and tips (bottom) regime. Green and black lines correspond to the measured CPS and the fitted envelope function. Red and blue curves correspond to Au 4f (5/2 left, 7/2 right) with oxidation states of 0 and +1, respectively, indicating a significant decrease in the Au^1+^/Au^0^ ratio for metal tips compared to SAC. Purple curves correspond to Zn 3p (1/2 left, 3/2 right), showing the increase in Au/Zn ratio for metal tips. (b) Au/Zn (blue) and Au^1+^/Au^0^ ratio (red) versus the Au/NC feed ratio, obtained from the XPS spectra of ZnSe-Au HNPs (see Figure S10). The Au/Zn ratio increases linearly with the Au/NC feed ratio, indicating a direct connection between the Au concentration in the metal deposition reaction and the final Au loading in the HNPs. The Au^1+^/Au^0^ ratio decreases significantly upon increasing Au loadings, proportional to (Au/NC)^−0.5^. This demonstrates the more ionic nature of the SAC regime, with more than 50% Au^1+^ for 50 Au/NC feed ratio, and the transition to the mostly metallic metal-tip regime, with less than 20% Au^1+^ for a 1000 feed ratio.

In order to obtain further insight into the chemical properties of the photocatalytic HNPs, and specifically the Au sites in the transition between the SAC and metal-tip regime, we conducted XPS measurements on ZnSe-Au HNPs with varying Au loadings (XPS analysis for all relevant elements is available in the Supporting Information, Figures S10–S12). Figure 3a shows normalized XPS spectra of ZnSe-Au HNPs in the SAC (top) and tips (bottom) regimes for the region corresponding to Au 4f and Zn 3p. The fit includes contributions of the Au^0^ oxidation state corresponding to metallic Au (red curves), and ionic Au^1+^ oxidation state (blue curves). The purple curves correspond to the Zn 3p.

Comparing the two spectra, we observed a significant increase in the Au/Zn ratio for metal tips compared to SACs. This is expected for the higher Au/NC feed ratio in the Au deposition reaction. Additionally, we observed a large Au^1+^/Au^0^ ratio for SACs, consistent with Au bound to Se rendering it with ionic character. There is then a significant decrease in the Au^1+^/Au^0^ ratio upon an increased feed ratio, consistent with the emergence of metallic Au species for higher Au loadings.

Beyond these two distinct cases of SACs and tips, we analyzed the XPS spectra of HNPs with varying Au loadings to characterize the transition between the two regimes in a quantitative manner (Figure S10). The results are summarized in Figure 3b, showing the Au/Zn (blue) and Au^1+^/Au^0^ ratios (red) versus the Au/NC feed ratio. The quantitative analysis yields a linear increase of the Au/Zn ratio with the Au/NC feed ratio, indicating a direct connection between the Au concentration in the metal deposition reaction and the final Au loading in the HNPs. Note that considering the dimensions of the NRs, the nominal surface coverage of Au at 300 Au/NC feed ratio corresponds to ∼0.5 Au/Zn among the surface atoms. At the higher loadings Au atoms deposit on existing Au sites forming tips.

At the same time, the Au^1+^/Au^0^ ratio decreases significantly with increasing Au loadings, demonstrating the change in the chemical nature of the Au sites throughout the transition from SACs to tips. For a 50 Au/NC feed ratio, the XPS analysis indicates more than 50% Au^1+^, showcasing the more ionic nature of the SAC regime. For higher Au loadings, the Au^1+^/Au^0^ decreases, proportional to (Au/NC)^−0.5^. Eventually, for a 1000 Au/NC feed ratio, the XPS analysis yields less than 20% Au^1+^. Considering in addition the enhanced surface sensitivity of the XPS, this indicates the dominant metallic nature of the metal-tip regime. These results agree with the XAFS analysis, and shed light on the nonmonotonic behavior of the ZnSe-Au HNPs as photocatalysts, which is related not only to the amount of deposited Au, but also to the photochemical reactivity of the Au sites.^7,61,68^

### Mechanistic Insights

An additional key feature essential for understanding the photoinduced catalytic reaction in the HNPs is the charge carrier dynamics and, in particular, the transfer of electrons to the metallic sites upon light absorption by the semiconductor. To elucidate the charge carrier dynamics in the novel SAC-HNPs, we performed ultrafast TA measurements, comparing HNPs in the SAC and metal-tip regime as well as pristine ZnSe NRs.

**Figure 4:**
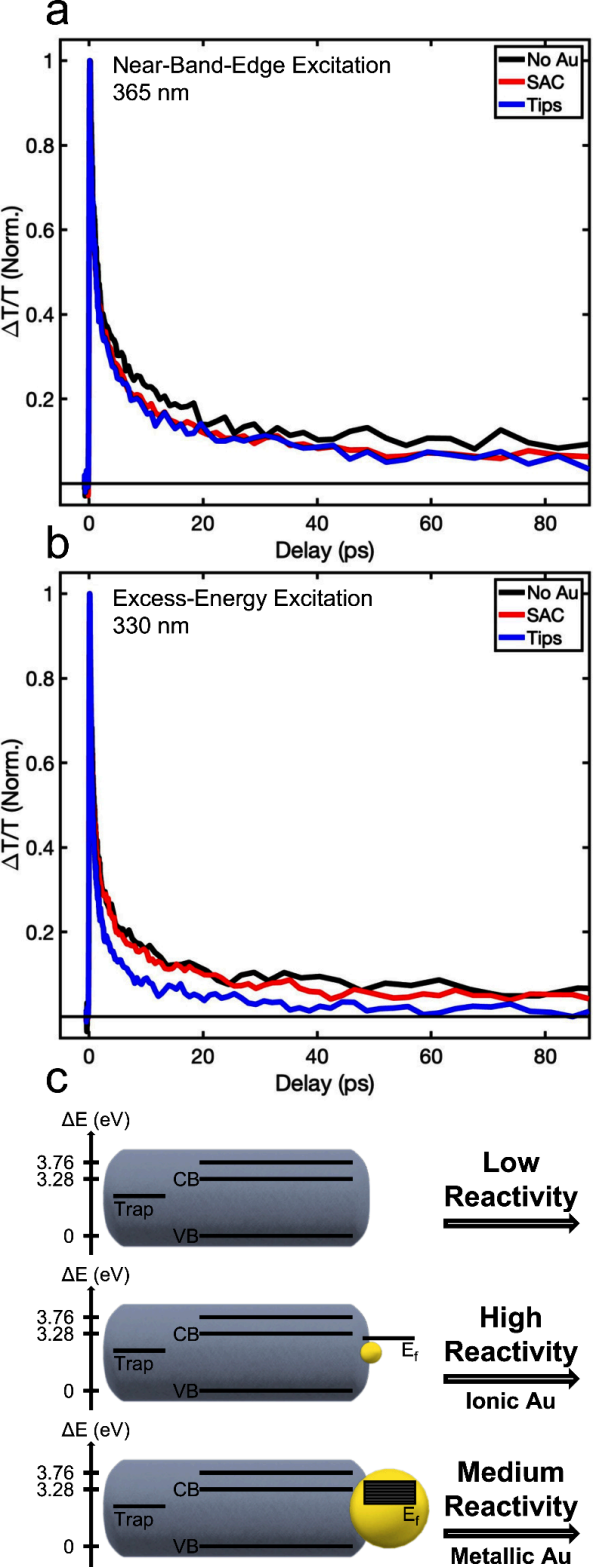
Ultrafast transient absorption. (a) Normalized TA dynamics of the bleach recovery at 380 nm, corresponding to the first excitonic transition of the ZnSe NRs, upon near-band-edge excitation at 365 nm (3.40 eV). The pump photon energy corresponds to the excitation energy used for the photocatalytic measurements. Black, red, and blue lines correspond to pristine ZnSe, ZnSe-Au SAC-HNPs (100 Au/NC), and Au-tipped NRs (1000 Au/NC), showing slightly faster recovery for Au-ZnSe, and no significant change in the charge carrier dynamics between the SAC and tips regimes. The long-lived component (>80 ps) and the short lifetimes (<5 ps) indicate a trapping mechanism, governing the charge carrier dynamics for near-band-edge excitation. (b) Normalized TA dynamics at 380 nm upon excess-energy excitation at 330 nm (3.76 eV). Whereas pristine ZnSe (black) and SAC-HNPs (red) show only minor changes upon increasing pump photon energy, the Au-tipped ZnSe (blue) reaches faster and complete bleach recovery, indicating a more efficient hot electron transfer to the Au tips. (c) Schematic representation of the charge carrier dynamics for ZnSe NRs (top) and ZnSe-Au HNPs in the SAC (middle) and metal-tip (bottom) regime. Upon near-band-edge excitation, the band-edge electron dynamics are mainly governed by traps for both pristine and Au-decorated ZnSe. Upon excess-energy excitation, hot electrons transfer more efficiently to the Au tips due to increasing density of states and lower Fermi level of the Au, both depending on the metal-tip size.^28^ For SAC-HNPs, metal decoration moderately affects the electron dynamics, with insignificant contributions to hot electrons dynamics. However, photocatalytic hydrogen formation, measured under near-band-edge excitation, is governed by the reactivity of the catalytic site. Deposition of Au as a catalytic site for the reduction reaction increases the QY of ZnSe NRs, with a lower reactivity for the metallic tip regime and significantly higher reactivity for the ionic SAC regime.

First, we studied the dynamics upon near-band-edge excitation at 365 nm (3.40 eV), corresponding to the excitation energy used for the photocatalytic study. Figure 4a presents the normalized TA dynamics of bleach recovery at 380 nm, corresponding to the first excitonic transition of the ZnSe NRs. The black, red, and blue lines correspond to pristine ZnSe, ZnSe-Au SAC-HNPs (100 Au/NC), and Au-tipped NRs (1000 Au/NC), respectively. Notably, pristine ZnSe NRs exhibit extremely fast bleach recovery compared to reported Cd-based NCs, with a lifetime of ∼3 ps. This observation is in agreement with previous TA studies on ZnSe nanostructures, attributing the fast bleach recovery to rapid trapping of electrons by surface defects, occurring on ps time scales.^69−72^

As for the Au-decorated NRs, both SAC-HNPs and tipped NRs exhibit even faster recovery than pristine ZnSe, demonstrating slightly improved charge separation consistent with the transfer of electrons to the Au sites. Interestingly, no significant change in the charge carrier dynamics was observed between the SAC and metal-tip regimes. This suggests that even upon Au growth, the fast trapping of charge carriers, seen also in the pristine ZnSe NCs, still governs the dynamics under near-band-edge excitation. Additional indications of that are the long-lived component (>80 ps) and the very short lifetimes (<3 ps), which are typical for trapping mechanisms governing the charge carrier dynamics.^28,64,69−71^

This observation provides mechanistic insight into the photocatalytic hydrogen formation, which was also measured under near-band-edge excitation. Reiterating, the TA data at 365 nm excitation show that the charge carrier dynamics was not affected by the metal site size, implying that the charge separation is not the rate-determining step in this system. On the other hand, XPS and XAS clearly show the unique coordination chemistry and chemical nature of SAC Au, differing from the metal-tip regime. Namely, the SAC regime comprises exclusively Au–Se bonds and manifests a dominant ionic Au character. Such characteristics endow the Au sites with higher reactivity.^36,37^ Thus, we conclude that the higher chemical reactivity of the SAC sites is the dominant factor determining their enhanced overall photocatalytic efficiency compared to tips.

For further insight into possible differences between the SAC and metal-tip regimes, we studied the dynamics at 380 nm upon excitation at 330 nm (3.76 eV), with excess energy above the band gap (Figure 4b). For pristine ZnSe (black) and SAC-HNPs (red), we observed only minor changes upon increasing the pump photon energy. However, the Au-tipped ZnSe (blue) reaches faster and complete bleach recovery. We assign this to a more efficient hot electron transfer to the Au tips. This is also shown in Figure S13, comparing the dynamics of each sample upon using different excitation energies.

The dynamics reflected in Figure 4a and b are in agreement with previous studies of HNPs in the metal-tip regime, showing more efficient electron transfer to larger metal sites. This is due to the increased density of states and lower Fermi level of the Au, both depending on the tip size.^2,28,73^ Accordingly, for SAC-HNPs, the discrete atomic-like density of states affects the electron dynamics only slightly, with almost negligible contributions even to hot electron dynamics. In contrast, the Au tips show efficient hot electron transfer, evident in faster and full bleach recovery. In our system, the effect of the metal site size on the charge carrier dynamics is observed only for hot electrons, with sufficient energy to overcome the surface trapping process, which is typical for ZnSe NCs.

Summarizing these mechanistic insights, Figure 4c shows a schematic representation of the charge carrier dynamics for ZnSe NRs (top) and ZnSe-Au HNPs in the SAC (middle) and metal-tip (bottom) regime.

## Conclusions

In this work, we developed and studied photocatalytic heavy-metal-free HNPs crossing over from the SAC to the metal-tip regime. The HNPs photocatalytic activity depends strongly on both the quantity and the nature of the metal sites, demonstrated by the nonmonotonic dependence of the QY on the Au/NC feed ratio. The HNPs in the SAC limit manifest optimal efficiency and high specific QY for photocatalytic hydrogen generation. These decrease upon the formation of metal tips. Therefore, optimal photocatalytic efficiency is achieved when the surface of the NCs is saturated with deposited SACs, yet below the critical loading from which metal clusters or tips emerge. This allows high photocatalytic performance for very low amounts of cocatalyst, which is beneficial for future applications.

In parallel to the photocatalytic study, we performed extensive structural analysis, including HAADF-STEM, XAS, and XPS. These complementary techniques were utilized to validate and characterize the new SAC regime and the transition between SAC and the metal-tip regime. Moreover, we explored the nature of the Au sites by integrating the structural and chemical analyses, revealing a gradual transition from “single-atoms” of ionic nature to metallic Au tips. Correlated with the photocatalytic performance of the corresponding HNPs, this suggests a significant contribution to the chemical reactivity of the metal sites.

To further address the effect of the metal domain regime on photocatalytic functionality, we studied the dynamics of the photoexcited charge carriers in the HNPs via ultrafast TA experiments. Upon near-band-edge excitation, corresponding to the conditions of the photocatalytic experiments, we measured similar charge transfer kinetics for the SACs and tips. In the case of excess-energy excitation, we observed hot electron transfer to the Au tip sites, revealing the effect of traps on the charge carrier dynamics. However, in the absence of excess energy, we conclude that the photocatalytic efficiency of the HNPs is determined by the chemical reactivity of the metal sites, which is superior in the case of the SACs.

In conclusion, HNPs in the SAC regime show promise toward efficient and sustainable heavy-metal-free photocatalysts, using a minimal amount of cocatalyst for maximal photocatalytic yield. The structural and chemical analyses, correlated with the photocatalytic and mechanistic studies, provide guidelines for the design of efficient, cost-effective, and environmentally compatible HNP-based photocatalysts for various applications.

## Experimental Section

### Chemicals

Zn acetate dihydrate (≥98.0%), 1-dodecanethiol (DDT, ≥98%), Se powder, AuCl_3_ (99%), oleylamine (OAm, 98%), polyethylenimine branched—*M*_w_ ∼ 25,000 (PEI), and l-ascorbic acid (99%) were purchased from Sigma-Aldrich. Oleylamine (OAm, 80–90%) was purchased from Thermo Scientific. Acetone was purchased from Gadot. Acetonitrile, toluene, and chloroform were purchased from Bio-Lab. All chemicals were used as received without further purification.

### Synthesis of ZnSe NRs

ZnSe NRs were synthesized via a previously reported procedure with minor modifications.^27^ Zn acetate dihydrate (1.5 g), OAm 80–90% (36 mL), and DDT (5 mL) were mixed in a 150 mL three-necked flask, heated under vacuum to 110 °C and degassed for 1 h. After cooling down to room temperature, the Zn solution was transferred to another flask containing Se powder (380 mg) and degassed at room temperature for 15 min. Then, the mixture was heated to 110 °C under Ar for 1 h to form ZnSe clusters. Next, the temperature was raised to 220 °C for 1 h to obtain ZnSe nanowires. Finally, the solution was heated to 270 °C for 20 min to obtain ZnSe NRs via Oswald ripening. After cooling to room temperature, the product was precipitated twice by centrifugation with acetone and acetonitrile and redispersed in toluene. Finally, the NRs were centrifuged with no antisolvent to obtain a clear dispersion of the ZnSe NRs in toluene. The dispersion was kept in the dark under an inert atmosphere for further use.

### Synthesis of ZnSe-Au HNPs

The NR stock solution was prepared by diluting the clean NRs-toluene dispersion to a concentration of 3 μM (4.5 nmol per sample). Au stock solution was prepared by mixing AuCl_3_ (10 mg), OAm 98% (150 molecules per Au atom), and toluene, followed by sonication at 50–60 °C until the red solution turned light yellow. To form HNPs with varying Au/NC feed ratios, the Au stock solution was diluted to the desired Au/NC molar ratios and mixed with NRs stock solution (1:1 volume) for 1 h in the dark under ambient atmosphere. The product was precipitated twice by centrifugation with acetone and redispersed in chloroform (1 μM).

### Sample Characterization

UV–vis absorbance spectra were obtained in solution by using a Jasco V-570 UV–vis–NIR spectrophotometer. STEM imaging was done utilizing Themis Z aberration-corrected STEM (Thermo Fisher Scientific) operated at 300 kV and equipped with a high-angle annular dark-field (HAADF) detector. The samples were prepared by drop-casting from solution onto an ultrathin carbon film supported by a lacey carbon film on a 400 mesh Cu grid (purchased from Ted Pella). XAFS data were collected at the NSLS-II synchrotron at Brookhaven National Laboratory, beamline 7-BM (QAS). The data were collected in fluorescence mode for Au L_3_-edge and in transmission mode for Zn and Se K-edges. The samples were prepared by drop-casting from solution onto Kapton film (1 mil) under an inert atmosphere and sealed using Kapton tape (1 mil). The details on the data analysis are given in the Supporting Information. XPS was measured using an Axis Supra instrument with monochromatic Al Kα (1486.7 eV). The samples were prepared by drop-casting from the solution onto a Si substrate.

### Ligand Exchange

PEI solution was prepared by dissolving PEI (*M*_w_ ∼ 25,000) in chloroform (0.15 g/mL). The PEI solution was mixed with the HNPs-chloroform dispersion (1:1 volume) for 1 h. Then, the product was precipitated twice by centrifugation with cyclohexane and redispersed in triple-distilled water (TDW). Finally, the HNPs were centrifuged with no additives to obtain a clear dispersion of ZnSe-Au in TDW.

### Hydrogen Formation Measurements

The hydrogen generation rate was measured using a gas chromatograph (GC, Varian 6820) equipped with a molecular sieve (5 Å) packed column and a thermal conductivity sensor. Two mL of aqueous solution of HNPs (0.5 OD at 365 nm) and ascorbic acid (0.2 M) were placed in a quartz cuvette, purged with Ar, and stirred while illuminated by a 365 nm LED (30 mW/cm^2^). Aliquots of the reaction vessel headspace were taken every 10 min using an automated gastight valve and quantified using the GC. See the Supporting Information for further details on the hydrogen formation measurements and the calculation of the quantum yield.

### Ultrafast Transient Absorption Measurements

Ultrafast TA measurements were performed with a home-built setup (see the Supporting Information). The differential transmission (Δ*T*/*T*) was measured as a function of the pump–probe delay for each probe wavelength. The pump and probe pulses were spatially overlapped on the sample at magic-angle (54.7°) polarization. In order to avoid any possible sample degradation during measurements, the signal levels of different TA scans were compared and no degradation was observed for the reported TA data.

## Supporting Information Available

Additional experimental details, including further structural characterization and mechanistic analysis (PDF)
