# Supplementary material for: Photocatalytic Semiconductor–Metal Hybrid Nanoparticles: Single-Atom Catalyst Regime Surpasses Metal Tips
Source: ACS Nano. 2025 Jan 6;19(2):2507–17. doi: 10.1021/acsnano.4c13603 (PMC11760151; doi:10.1021/acsnano.4c13603)
Supplement: Supplementary file 1 — nn4c13603_si_001.pdf [file nn4c13603_si_001.pdf]

Photocatalytic Semiconductor-Metal Hybrid Nanoparticles: Single-Atom Catalyst Regime Surpasses Metal Tips

Shira Gigi,[a,b] Tal Cohen,[a,b] Diego Florio,[c,d] Adar Levi,[a,b] David Stone,[a,b] Ofer Katoa,[a,b] Junying Li,[e] Jing Liu,[f] Sergei Remennik,[b] Franco V. A. Camargo,[d] Giulio Cerullo,[c,d] Anatoly I. Frenkel,[e,g] Uri Banin*[a,b]

[a] Institute of Chemistry, The Hebrew University of Jerusalem, Jerusalem 9190401, Israel
[b] The Center for Nanoscience and Nanotechnology, The Hebrew University of Jerusalem, Jerusalem 9190401, Israel
[c] Dipartimento di Fisica, Politecnico di Milano, Milano 20133, Italy
[d] Istituto di Fotonica e Nanotecnologie, Consiglio Nazionale delle Ricerche, Milano 20133, Italy
[e] Department of Materials Science and Chemical Engineering, Stony Brook University, Stony Brook, NY 11794, USA
[f] Department of Mathematics and Physics, Manhattan University, Riverdale, NY 10471, USA
[g] Chemistry Division, Brookhaven National Laboratory, Upton, NY 11973, USA

Table of Contents

Experimental Section **2**

Supplementary Data **3**

- UV-VIS Absorbance 3
- HAADF-STEM Characterization 4
- Photocatalytic Activity 5
- Catalyst Characterization After Photocatalytic Reaction 5
- XAFS Analysis 8
- XPS Analysis 11
- Ultrafast Transient Absorption 14

Experimental Section

XAFS Analysis: Data were processed and analyzed using the Athena and Artemis software packages.[1] The Athena software was needed to assign the photoelectron energy origin, E_0, and to perform edge-step normalization and background subtraction of the measured X-ray absorption coefficient data. The background-subtracted and edge-step normalized absorption coefficient data were then converted to k space. The k^2-weighted data were Fourier transformed (FT) to r-space, and EXAFS fitting was performed in r-space using Artemis. For Au-Au and Au-Se contributions, theoretical single-scattering paths were calculated using FEFF6 software.

Ultrafast Transient Absorption Measurements: Ultrafast transient absorption (TA) measurements were performed with a home-built setup seeded by a regeneratively amplified Ti:sapphire laser system (Libra, Coherent) emitting 100-fs pulses at 800 nm wavelength and 2 kHz repetition rate. The excitation pulses at 330 and 365 nm were generated by frequency doubling 10-nm bandwidth pulses at central wavelengths of 660 and 730 nm, respectively, which were generated using a home-made optical parametric amplifier. A 50 μm thick β-barium borate (BBO) crystal was used for second harmonic generation, followed by a UV filter to reject the remaining visible light. The broadband probe pulses (330-600 nm) were obtained by tight-focusing the fundamental wavelength pulses in a 3-mm-thick CaF_2 crystal. A BG39 filter was used to reject the remaining light at 800 nm. After the sample the transmitted probe was sent to a spectrometer (SP2150 Acton, Princeton Instruments) and detected using a linear image sensor driven and read out by a custom-built board (Stresing Entwicklungsburo). The differential transmission ($\Delta T/T$) was measured as a function of the pump-probe delay for each probe wavelength. The pump and probe pulses were spatially overlapped on the sample at magic-angle (54.7°) polarization. In order to avoid any possible sample degradation during measurements, the signal levels of different TA scans were compared, and no degradation was observed for the reported TA data.

Supplementary Data

UV-VIS Absorbance

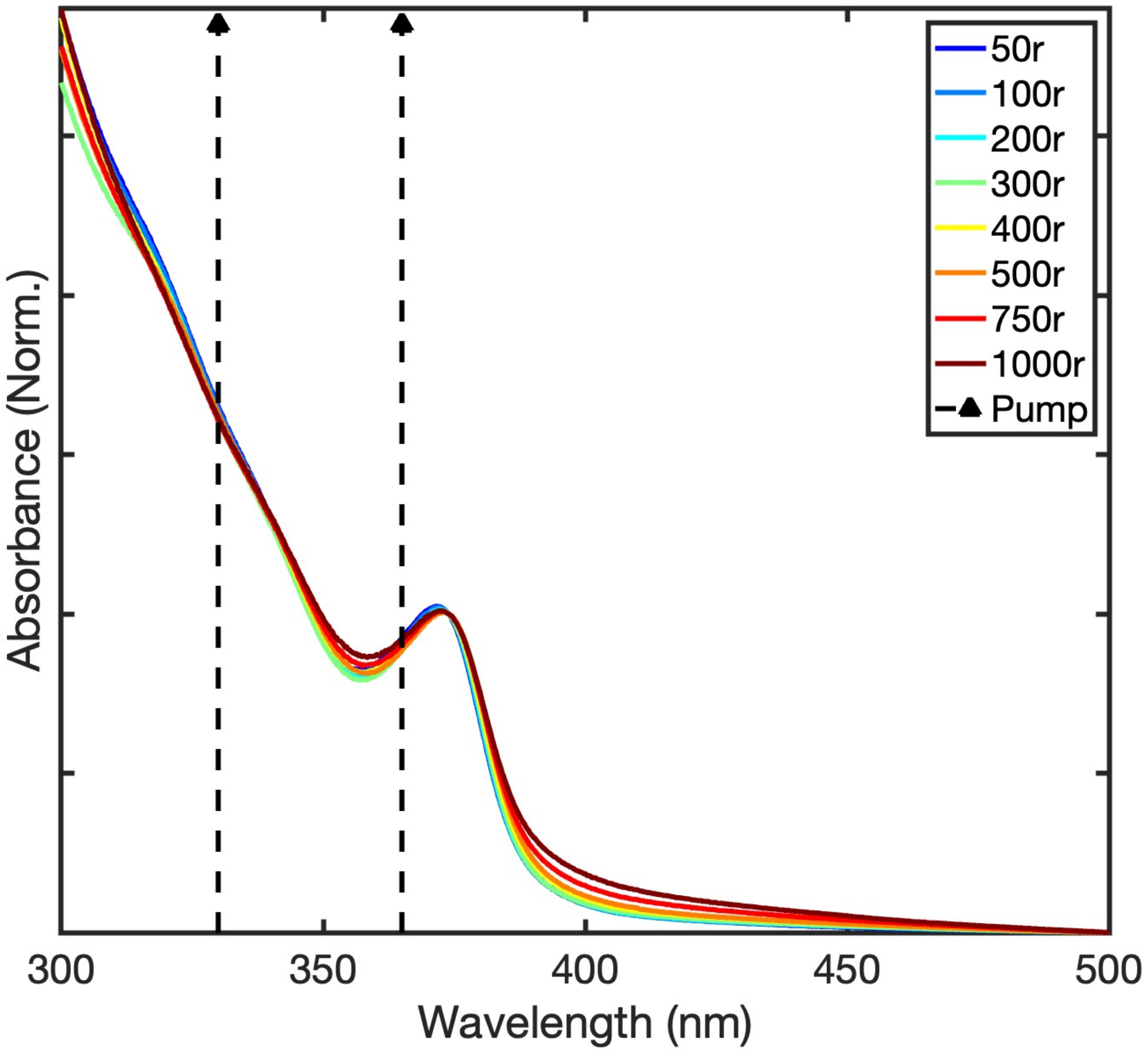

Figure S1. Normalized absorbance spectra of ZnSe-Au HNPs, ranging from 50 Au/NC feed ratio (blue) to 1000 Au/NC feed ratio (brown). The transition from SAC (50r-300r) to metal tips (300r-1000r) results in a gradual redshift and broadening of the spectra, attributed to the formation of Au clusters. At the excitation wavelength used in the photocatalytic study (365 nm), the spectral changes due to Au growth are less than 5%. Vertical dashed lines indicate the pump photon energies used in the TA experiments, with near-band-gap excitation at 365 nm and excess-energy excitation at 330 nm.

HAADF-STEM Characterization

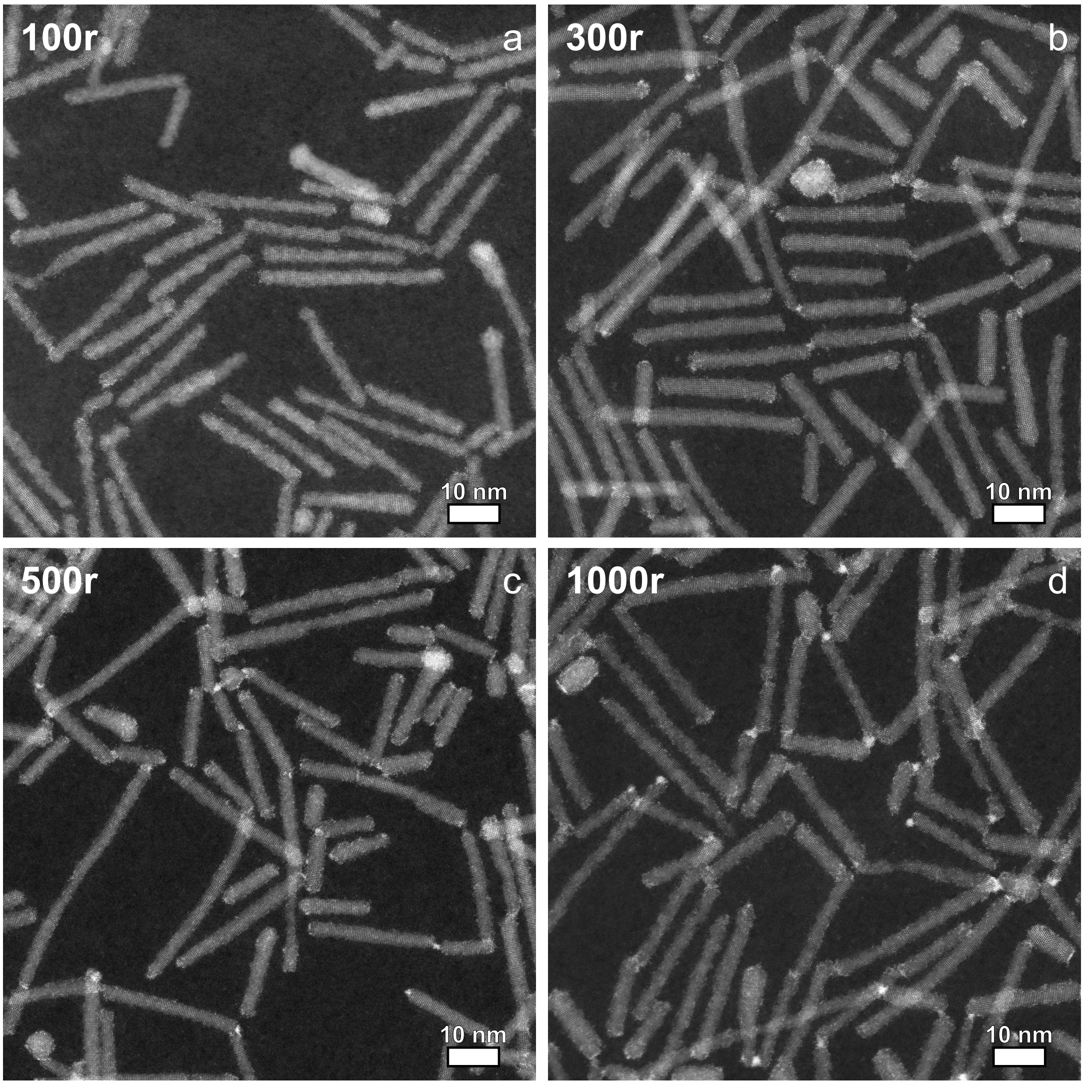

Figure S2. HAADF-STEM images of ZnSe-Au HNPs synthesized with different Au/NC feed ratios (100, 300, 500, and 1000), showing the gradual transition from a) SACs to d) metal tips with increasing Au loading. Au sites appear as bright spots on the NRs surface.

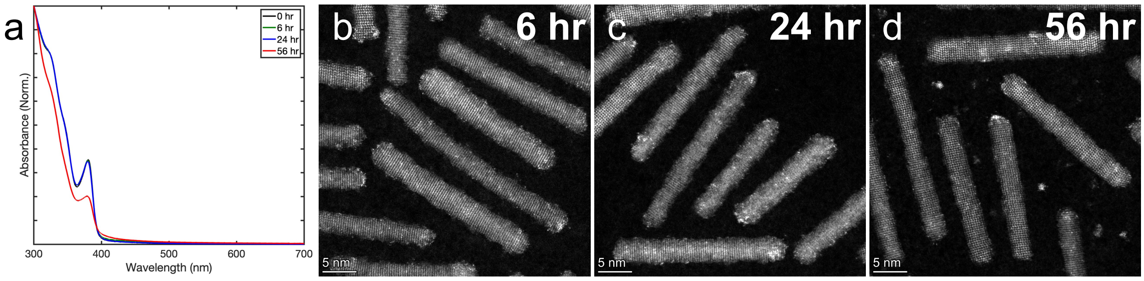

Figure S3. Stability experiments. a) Absorbance spectra and b-d) HAADF-STEM images of ZnSe-Au HNPs in the SAC regime after heating to 80 °C under ambient atmosphere. b) After 6 hours, no structural or spectral changes were observed (green line in panel a), as the SACs retain their homogeneous dispersion on the ZnSe surface. c) After 24 hours, sintering of Au atoms occurred, forming sub-nanometric Au clusters. However, most Au atoms remained isolated and homogeneously dispersed, and no spectral change was observed (blue line in panel a). d) After 56 hours, the Au atoms formed small tips, followed by redshift and spectral broadening (red line in panel a). By that stage, the HNPs had started to disassemble, as both Au and ZnSe clusters were detached from the surface due to the prolonged heating.

Photocatalytic Activity

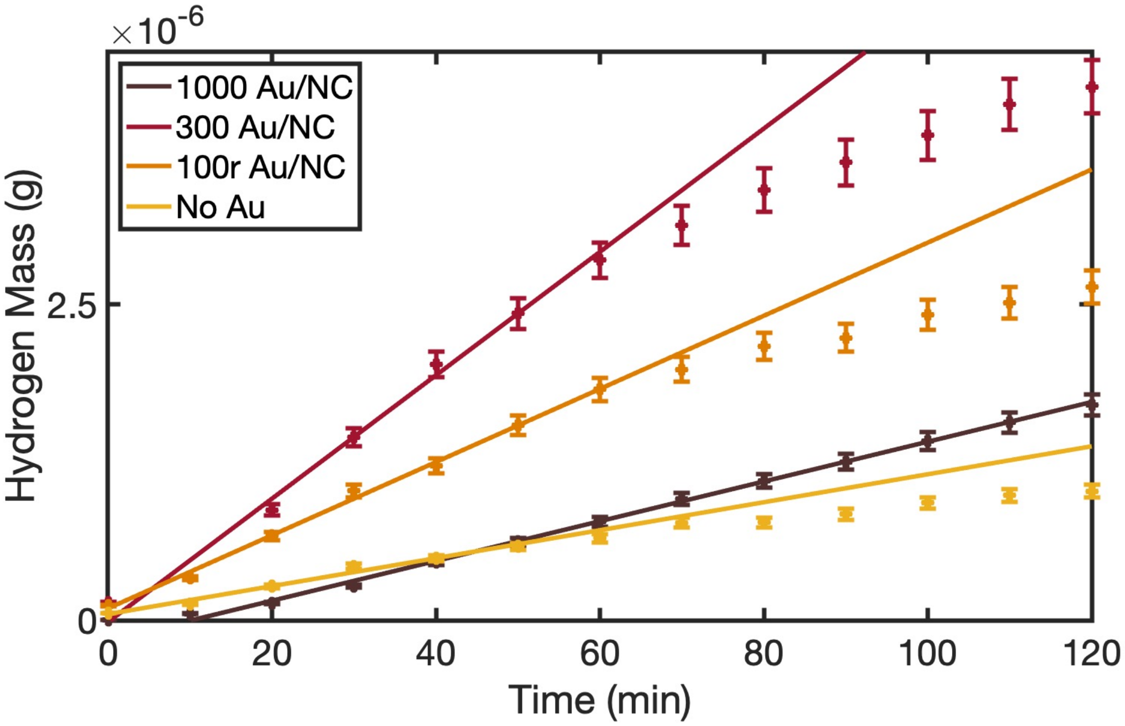

Figure S4. Hydrogen evolution over time for pristine ZnSe (yellow line) and ZnSe-Au HNPs with 100, 300, and 1000 Au/NC feed ratio (orange, red, and brown lines, respectively). Linear fitting of the hydrogen evolution rate during the first hour was used to calculate the photocatalytic quantum yield according to Eq. (1). The first point of 1000 Au/NC (brown line) is omitted from the linear fit due to the delay in hydrogen gas detection related to its saturation in solution.

The measured hydrogen evolution rates we used to calculate the photocatalytic quantum yield (QY):

(1) $$QY = \frac{n_e}{n_p} \cdot 100\% = \frac{2n_{H_2}}{n_p} \cdot 100\%$$

where $\boldsymbol{n_e}$ is the number of electrons participating in the reduction reaction, $\boldsymbol{n_p}$ is the number of photons absorbed by the HNPs, and $\boldsymbol{n_{H_2}}$ is the number of hydrogen molecules formed, as detected by the GC. $\boldsymbol{n_p}$ was calculated based on the illumination intensity, the HNPs absorption, and the irradiation time.
The specific QY of the Au atomic sites was calculated by:

(2) $$QY_{specific} = \frac{QY - QY_{ZnSe}}{(Au/Zn)} = \frac{QY - QY_{ZnSe}}{8 \cdot 10^{-5} \times (Au/NC)}$$

where QY_{ZnSe} is the QY of pristine ZnSe (with no Au), and the Au loading in the HNPs is represented by the Au/Zn ratio in the sample. As shown by the XPS analysis (Figure 3b), the Au/Zn ratio is linearly dependent on the Au/NC feed ratio, with a proportionality constant of $8 \cdot 10^{-5}$.
The photocatalytic hydrogen formation experiments were performed in aqueous solutions, with ascorbic acid (0.2 M) as a hole scavenger. Similar experiments with ethanol as the hole scavenger resulted in poor performance of the ZnSe-Au HNPs, regardless of the Au loading or regime.

Catalyst Characterization After Photocatalytic Reaction

In order to examine the stability of the HNPs during the photocatalytic reaction, we characterized their structure after 30 minutes of illumination under full working conditions, i.e., in aqueous solution containing ascorbic acid (0.2 M), under 365 nm illumination (30 mW/cm^2), and under Ar atmosphere. Figure S5 shows HAADF-STEM images of HNPs with a feed ratio of 100, 300, and 1000 after the photocatalytic reaction in water. For 100 Au/NC feed ratio (panels a-b), corresponding to the SAC regime, the Au sites retain their configuration as single Au atoms dispersed on the NRs surface, and no aggregation of Au is observed. For 300 Au/NC (panels c-d), corresponding to the highest Au loading in the SAC regime, small Au clusters are observed on some of the NRs, indicating partial aggregation of Au during the photocatalytic reaction. For 1000 Au/NC feed ratio (panels e-f), corresponding to the metal-tip regime, the Au sites retain their original configuration as metal tips.

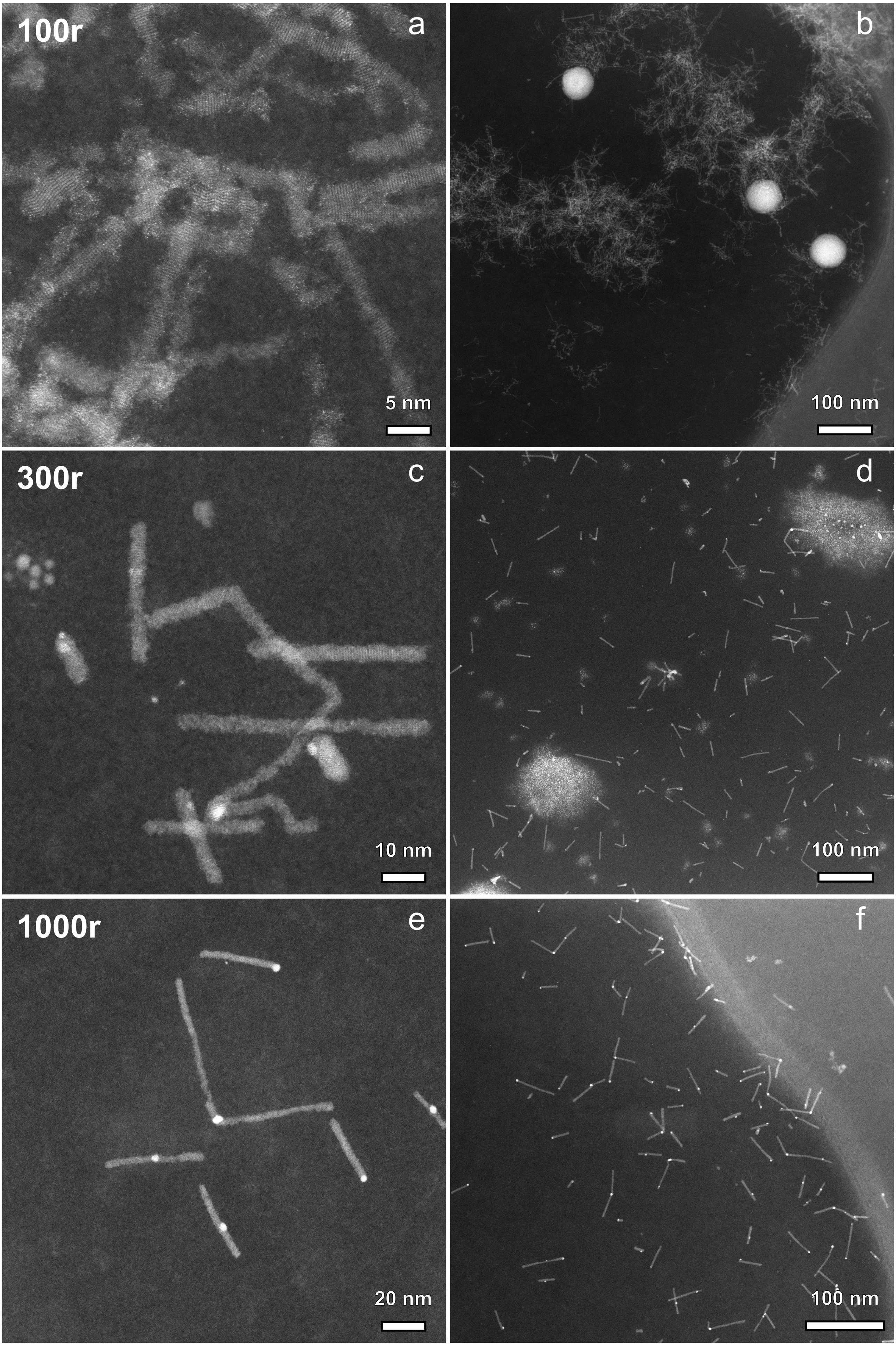
100r
a
5 nm
b
100 nm
300r
c
10 nm
d
100 nm
1000r
e
20 nm
f
100 nm

Figure S5. Stability experiments after photocatalytic reaction. HAADF-STEM images of ZnSe-Au HNPs with different Au loadings after photocatalytic hydrogen generation. a-b) For 100 Au/NC feed ratio, corresponding to the SAC regime, the single Au atoms retain their configuration, and no aggregation of Au is observed. c-d) For 300 Au/NC, which is the highest Au loading in the SAC regime, partial aggregation of Au is observed, as small Au clusters appear on some of the NRs. e-f) For 1000 Au/NC feed ratio, corresponding to the metal-tip regime, the Au tips retain their original configuration.

Noticeably, STEM imaging of water-based samples poses technical challenges resulting in lower resolution. Additionally, the long polymeric ligands (PEI, see Experimental Section in the main manuscript) further decrease the resolution obtained by STEM and impede the detection of atomic Au sites. In order to obtain further insight into the structural stability of the HNPs under working conditions, while still enabling high resolution imaging, we performed stability experiments also in organic medium. The HNPs were dispersed in toluene and illuminated (365 nm, 30 mW/cm^2) for 30 minutes under ambient atmosphere. Figure S6 shows the HAADF-STEM characterization of HNPs with 300 Au/NC feed ratio, corresponding to the highest Au loading in the SAC regime. Single Au atoms are clearly observed as bright spots on the NRs body. Additionally, small Au clusters appear on some of the NRs, indicating partial aggregation of the Au atoms, as was observed for this Au loading also under full working conditions (Figure S5, panels c-d). However, the partial aggregation occurred only for the highest SACs loading, whereas the 100 Au/NC feed ratio showed no clustering of Au even under full working conditions (Figure S5, panels a-b).

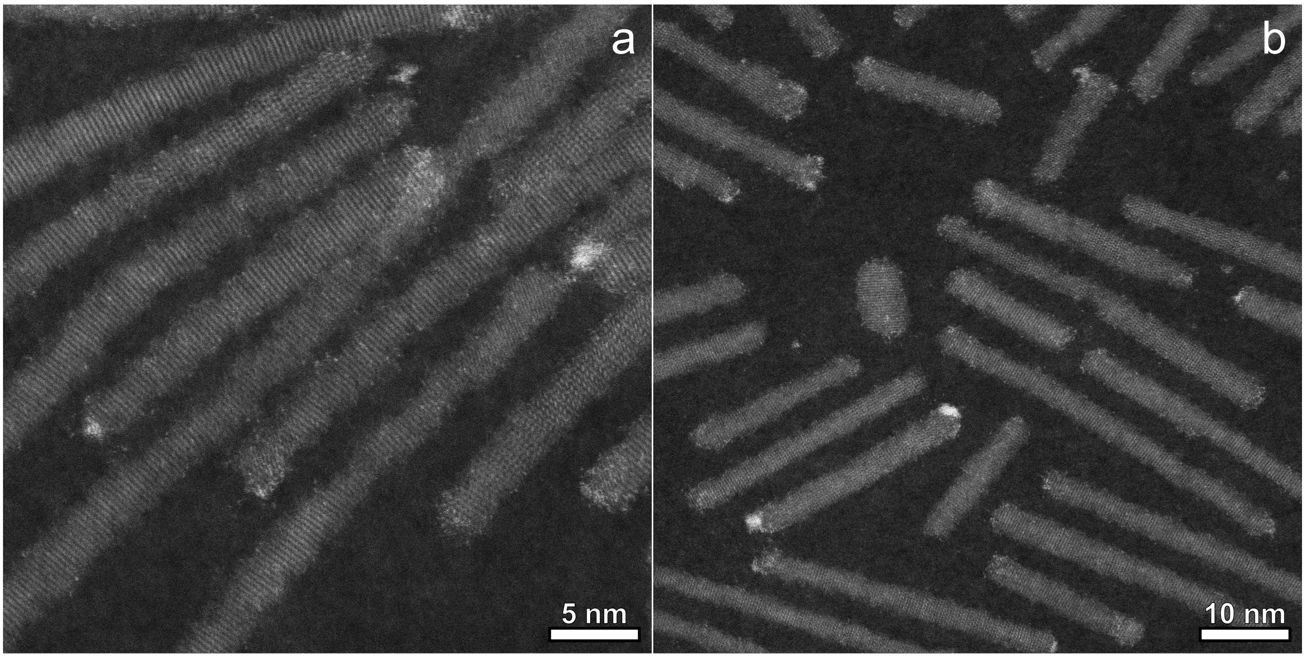

Figure S6. Stability experiment in organic medium. HAADF-STEM images of ZnSe-Au HNPs with a feed ratio of 300 Au/NC after 30 minutes of illumination in toluene under ambient conditions. The 300 Au/NC feed ratio corresponds to the highest Au loading in the SAC regime. The STEM images indicate dispersion of atomic Au sites on the NRs surface, alongside partial aggregation of Au to form small clusters on some of the NRs.

XAFS Analysis

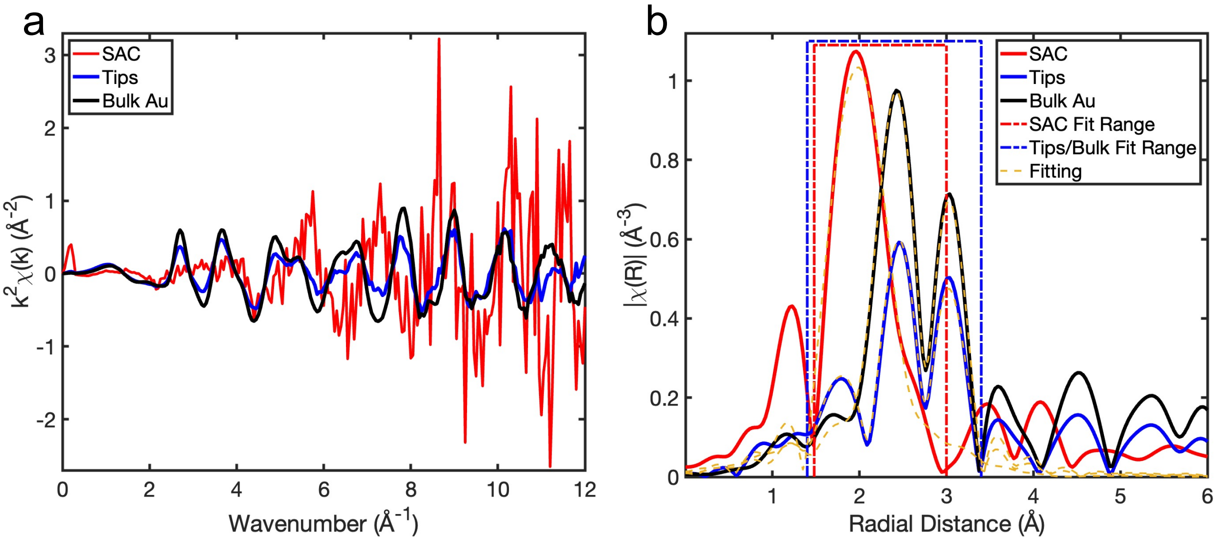

Figure S7. Au L_3-edge XAFS characterization. a) *k*-space and b) *r*-space spectra of ZnSe-Au HNPs in the SAC (red) and tips (blue) regime, compared to that of a reference Au foil sample corresponding to bulk Au (black). Yellow dashed lines show the fitted models. Red and blue dashed lines indicate the fitting range for SAC and for tips and bulk Au, respectively.

To obtain quantitative structural information about Au in the different regimes, XAFS analysis was performed using the Demeter package.[1] First, we analyzed the data collected for the Au foil reference sample to obtain the passive electron reduction factor (S_0^2). Then, the so obtained S_0^2 was used to fit the spectra of ZnSe-Au HNPs in the SAC and metal-tip regime. The fitting parameters are shown in Table S1.

Table S1. Fitting parameters of Au L_3-edge *r*-space data.

Sample	Path	CN	S_0^2	R (Å)	σ^2 (Å^2)	ΔE_0 (eV)
Bulk Au	Au-Au	12	0.81 ± 0.04	2.864 ± 0.003	0.0077 ± 0.0005	5.5 ± 0.3
Au SACs	Au-Se	2.86 ± 0.73	0.81[a]	2.39 ± 0.01	0.003 ± 0.002	-6.8 ± 3.4
Au Tips	Au-Se	1.31 ± 0.61	0.81[a]	2.40 ± 0.01	0.009 ± 0.004	-6.8[b]
	Au-Au	8.50 ± 0.76	0.81[a]	2.87 ± 0.01	0.008 ± 0.001	5.0 ± 0.5

[a] Obtained from the fitting of Au foil data corresponding to bulk Au. [b] Fixed to the value of Au SACs.

For SAC-HNPs, the fitted model indicates a single dominant contribution stemming from the Au-Se scattering path. The average coordination number for SAC-Au (2.86±0.73), which is lower than that of ZnSe and AuSe (CN=4), is attributed to a structure in which isolated Au atoms are deposited on the surface of the NRs, where each Au atom is bonded to approximately three Se atoms. However, for the case of HNPs in the metal-tip regime, two main scattering paths contribute to the *r*-space spectrum: Au-Se and Au-Au. In order to interpret the XAFS data, several possible structural models should be considered. First, we examine a structure in which two segregated Au phases coexist. The first phase consists of isolated Au atoms decorating the ZnSe surface, similar to the case of the SAC-HNPs. The second phase comprises small Au clusters (tips) deposited on the NRs, in which the vast majority of Au atoms are bonded to other Au atoms (Scheme S1, left). The average coordination number corresponding to the SAC phase (Table S1, 3rd entry) can be described as:

$$(3)\quad CN^{Au-Se} = \frac{n_{Au-Se}}{n_{Au}^{tot}} = \frac{n_{Au-Se}}{n_{Au}^{SAC}} \cdot \frac{n_{Au}^{SAC}}{n_{Au}^{tot}} = CN^{SAC} \cdot \frac{n_{Au}^{SAC}}{n_{Au}^{tot}}$$

where n_{Au-Se} is the total number of Au-Se nearest-neighbors pairs, n_{Au}^{tot} is the total number of absorbing Au atoms, and n_{Au}^{SAC} is the number of absorbing atoms in the SAC phase.[2] Notice that according to this model of two segregated Au

phases, CN^{SAC} can be derived from the fitting of SAC-HNPs (Table S1, 2nd entry). Therefore, we can calculate the fraction of Au atoms in the SAC phase by:

$$(4) \quad \frac{n_{Au}^{SAC}}{n_{Au}^{tot}} = \frac{CN^{Au-Se}}{CN^{SAC}} = 0.46 \pm 0.24$$

And the fraction of Au atoms in the tips phase is then:

$$(5) \quad \frac{n_{Au}^{Tips}}{n_{Au}^{tot}} = 1 - \frac{n_{Au}^{SAC}}{n_{Au}^{tot}} = 0.54 \pm 0.24$$

We can then apply a similar calculation to the tips phase:

$$(6) \quad CN^{Au-Au} = \frac{2n_{Au-Au}}{n_{Au}^{tot}} = \frac{2n_{Au-Au}}{n_{Au}^{Tips}} \cdot \frac{n_{Au}^{Tips}}{n_{Au}^{tot}} = CN^{Tips} \cdot \frac{n_{Au}^{Tips}}{n_{Au}^{tot}}$$

where n_{Au-Au} is the total number of Au-Au nearest-neighbors pairs, n_{Au}^{Tips} is the number of absorbing atoms in the tips phase, and CN^{Au-Au} is derived from the Au-Au component of the fitting (Table S1, 4th entry). Finally, we can derive the average coordination number of Au atoms in the tips phase:

$$(7) \quad CN^{Tips} = CN^{Au-Au} \cdot \left(\frac{n_{Au}^{SAC}}{n_{Au}^{tot}}\right)^{-1} = 16 \pm 7$$

The coordination number of Au in the tips is clearly overestimated, as the highest CN for any Au cluster is 12. Considering the margin of error, this result may agree with physical CN values. However, to properly interpret the XAFS data, this model should be carefully adjusted while correlated with the HAADF-STEM data. As shown in Figure 1c and S2d, the average tip size ranges between 1-2 nm, corresponding to coordination numbers of 7-9, using a model of supported clusters.[2,3] While these values are well within the margin of error, this model is not sufficient to explain the intricate structure of coexisting SACs and tips.

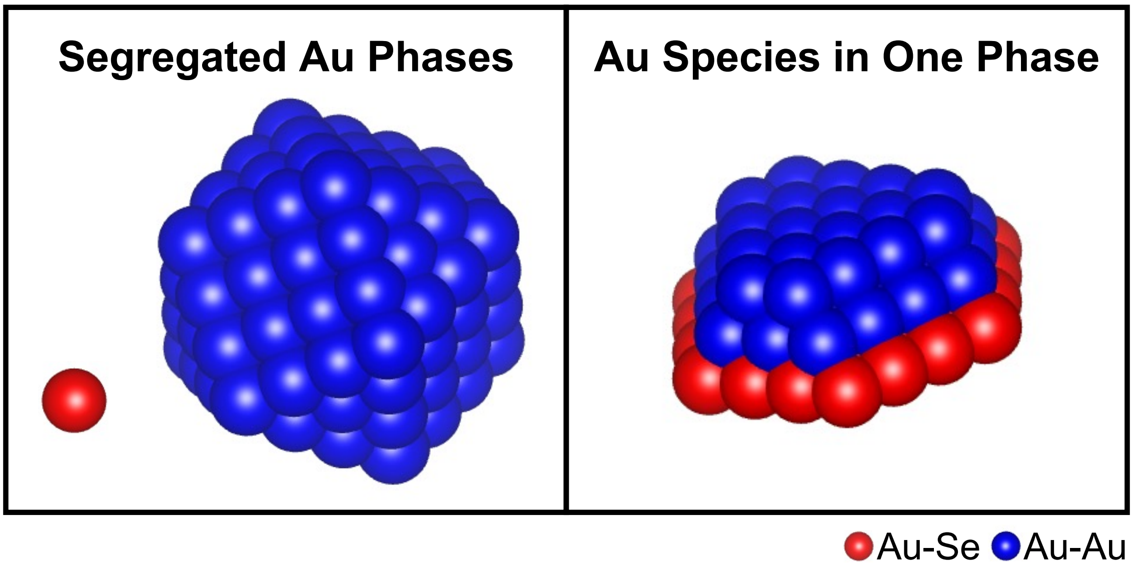

Scheme S1. Structural models of Au sites in the metal-tip regime. In segregated phases (left), Au-Se and the Au-Au species correspond to SACs and tips, respectively. In a single phase (right), Au-Se species correspond to Au atoms in the ZnSe-Au interface, whereas the Au-Au species include the Au atoms that are not in direct contact with the ZnSe surface. Illustrations were generated in VESTA.[4]

Nonetheless, a key assumption in the first model involves the coordination chemistry of the SAC phase. We attributed the Au-Se component in the metal-tips regime to isolated Au atoms decorating the ZnSe surface, just as in the SAC-HNPs sample. However, clustering of the "single" Au atoms is evident in the STEM images. Therefore, we propose a second model, in which all Au atoms sinter and form Au tips. Here, we assume only one phase (tips) comprising two Au species. The ionic species, corresponding to the Au-Se scattering path, includes only Au atoms in the ZnSe-Au interface. The metallic species, corresponding to the Au-Au path, includes all other Au atoms which are not in direct contact with the ZnSe surface (Scheme S1, right). The coordination number of the ionic species (Table S1, 3rd entry) can be described as:

$$(8) \quad CN^{Au-Se} = \frac{n_{Au-Se}}{n_{Au}^{tot}} = \frac{n_{Au-Se}}{n_{Au}^{ionic}} \cdot \frac{n_{Au}^{ionic}}{n_{Au}^{tot}}$$

where n_{Au}^{ionic} is the number of absorbing atoms in the ZnSe-Au interface. Assuming each Au atom in the interface is bonded to three Se atoms, the fraction of atoms attached to the ZnSe surface is:

$$(9)\quad \frac{n_{Au}^{SAC}}{n_{Au}^{tot}} = \frac{CN^{Au-Se}}{3} = 0.44 \pm 0.22$$

Since all absorbing Au atoms are in one phase, one commonly used model is a truncated (by the (111) plane) cuboctahedron Au cluster, in which approximately half of the atoms are in the bottom Au layer, i.e., attached to the ZnSe surface. Additionally, the diameter of the cluster should fit the average cluster size observed by STEM (1-2 nm). The best fit considering the aforementioned constrains is a truncated cuboctahedron of cluster order 3 (the number of spacings between the nearest atoms on the cuboctahedron edge), consisting of 3 Au layers (Scheme S1, right). Out of 82 atoms forming this cluster, the bottom layer comprises 37 atoms, corresponding to the desired fraction of ionic species. The calculated diameter is 1.7 nm, agreeing with the STEM data. Noticeably, this model does not take into account the coexistence of several Au phases, which can be seen in the HAADF-STEM. Figure 1c and S2d show a variety of Au structures, including isolated atoms, amorphous accumulation of atoms, and clusters of different size. Like the first model, this description does not paint a full picture of the structural properties of Au in the tipped NRs. Nevertheless, the two proposed models – segregated Au phases versus different Au species within the same phase – represent two extreme limits, spanning a whole range of possible Au dispersions. Evidently, careful analysis of the XAFS data (representing an ensemble-average result for coexisting clusters and "single atoms"), correlated with STEM (a local imaging technique that lacks sufficient statistical significance of XAFS, but provides a glimpse at the possible coexisting species) and XPS (providing another ensemble-average result on the distribution of oxidation states of Au species), is required to draw valid conclusions regarding the Au dispersion in the HNPs.

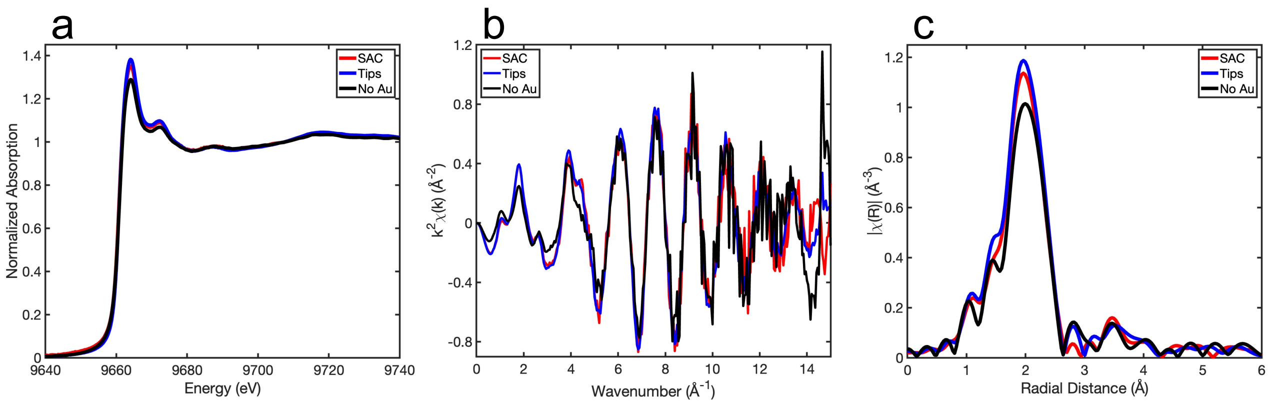

Figure S8. Zn K-edge XAFS characterization. a) Normalized XANES, b) *k*-space, and c) *r*-space spectra of ZnSe-Au HNPs in the SAC (red) and tips (blue) regime, compared to that of pristine ZnSe NRs (black). Zn K-edge shows no significant trend related to the presence of Au or to the Au loading, as expected for the host material considering the low amount of dopant.

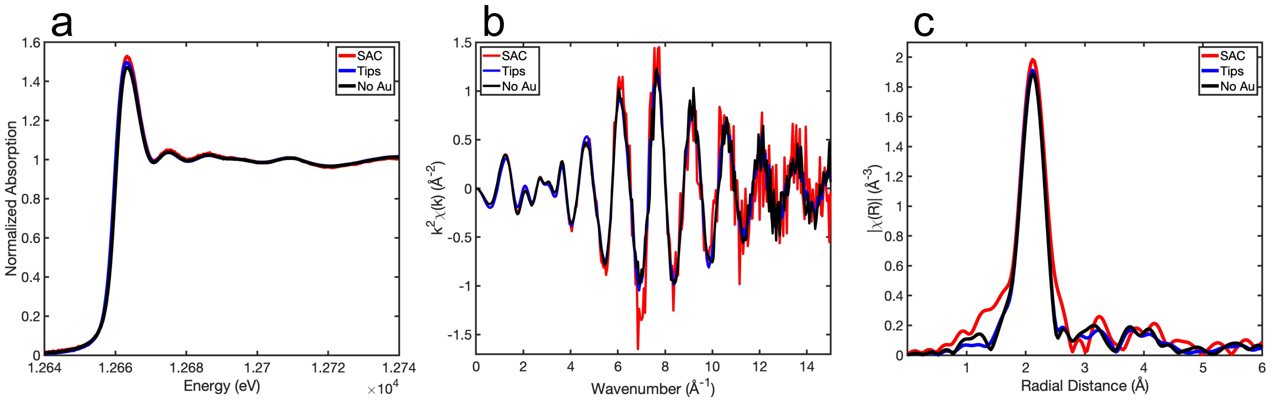

Figure S9. Se K-edge XAFS characterization. a) Normalized XANES, b) *k*-space, and c) *r*-space spectra of ZnSe-Au HNPs in the SAC (red) and tips (blue) regime, compared to that of pristine ZnSe NRs (black). Se K-edge shows no significant change related to the presence of Au or to the Au loading, as expected for the host material considering the low amount of dopant.

XPS Analysis

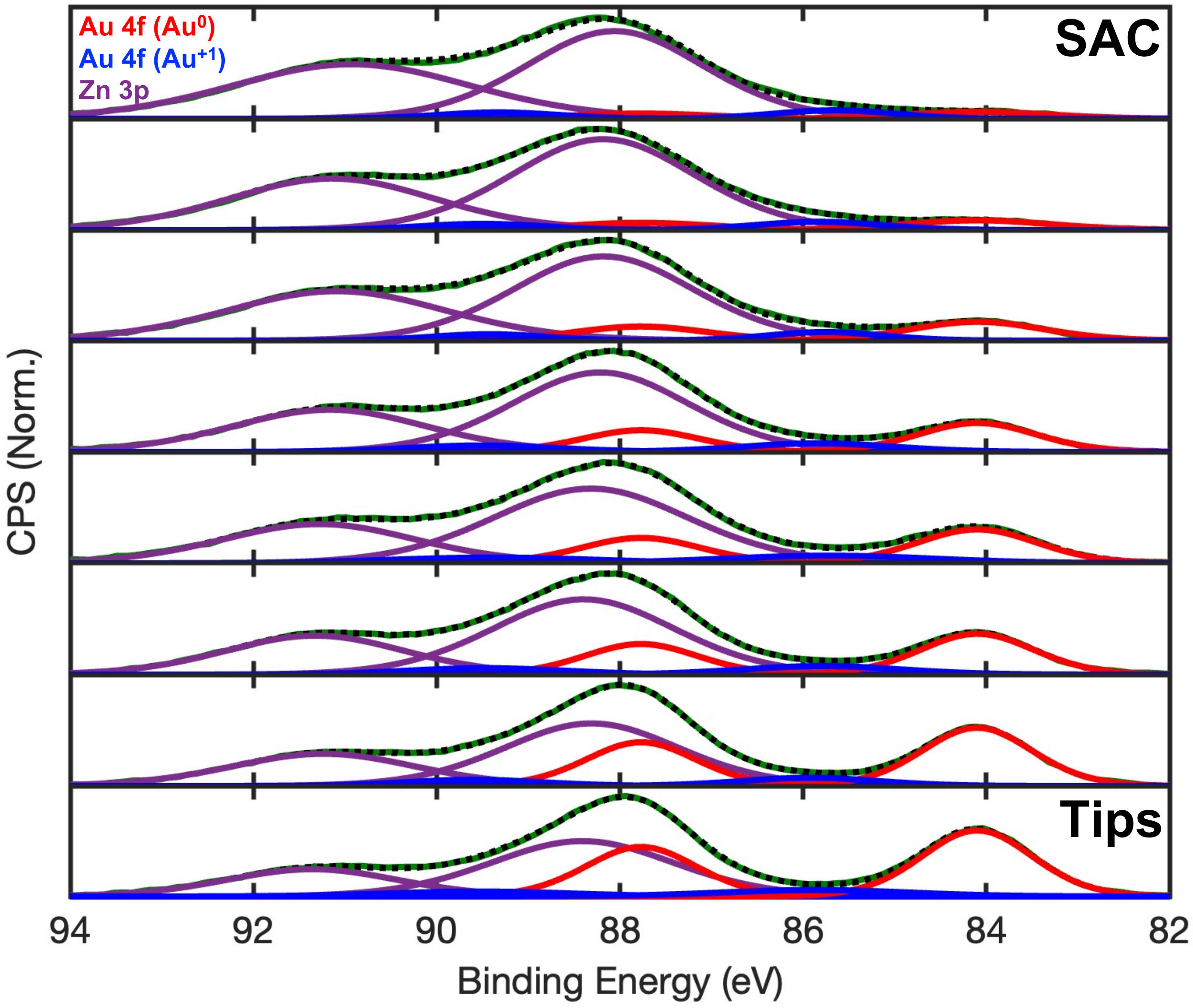

Figure S10. Normalized XPS spectra of ZnSe-Au HNPs with varying Au loadings, ranging from 50 (top) to 1000 (bottom) Au/NC feed ratio. Green and black lines correspond to the measured CPS and the fitted envelope function, respectively. Red and blue curves correspond to Au 4f (5/2 left, 7/2 right) with oxidation state of 0 and +1, respectively, indicating a significant decrease in the Au^{+1}/Au^{0} ratio for increasing Au loadings. Purple curves correspond to Zn 3p (1/2 left, 3/2 right), showing the increase in Au/Zn ratio for increasing Au/NC feed ratios.

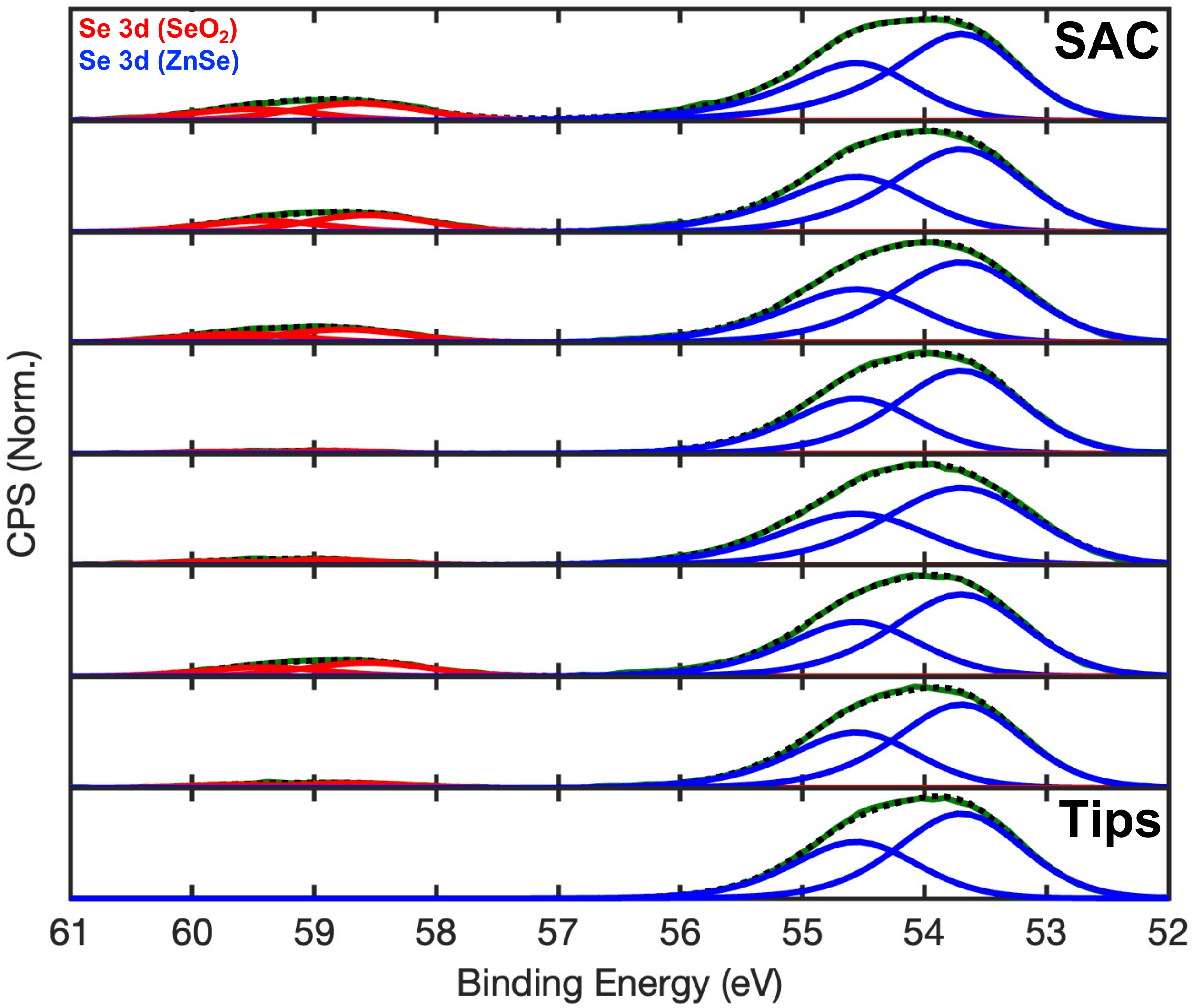

Figure S11. Normalized XPS spectra of ZnSe-Au HNPs with varying Au loadings, ranging from 50 (top) to 1000 (bottom) Au/NC feed ratio. Green and black lines correspond to the measured CPS and the fitted envelope function, respectively. Red and blue curves correspond to Se 3d from SeO_2 and ZnSe, respectively. The spectra show no clear trend related to the Au loading.

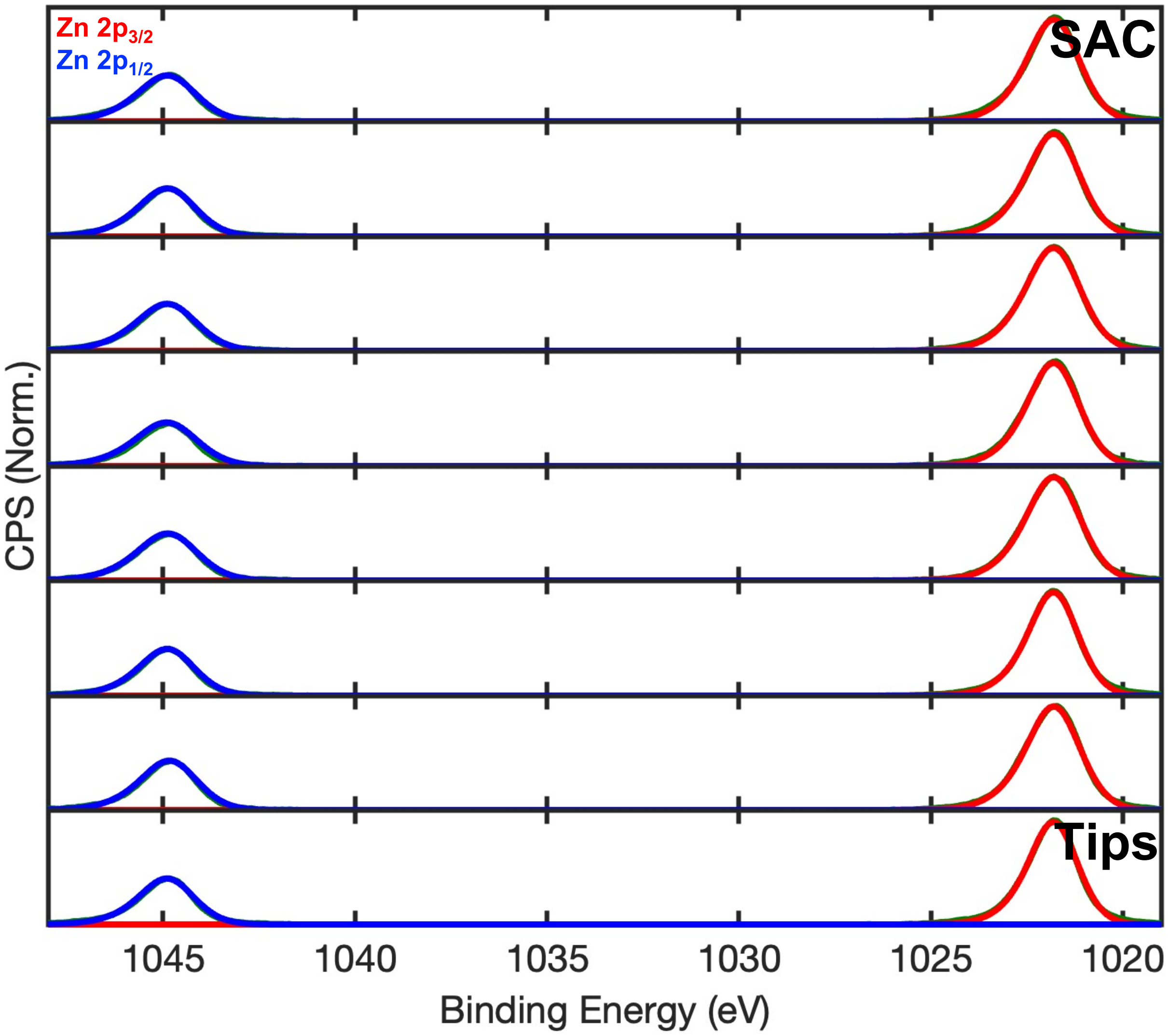

Figure S12. Normalized XPS spectra of ZnSe-Au HNPs with varying Au loadings, ranging from 50 (top) to 1000 (bottom) Au/NC feed ratio. Green and black lines correspond to the measured CPS and the fitted envelope function, respectively. Red and blue curves correspond to Zn 2p 3/2 and Zn 2p 1/2, respectively. The spectra show no change related to the Au loading.

Ultrafast Transient Absorption

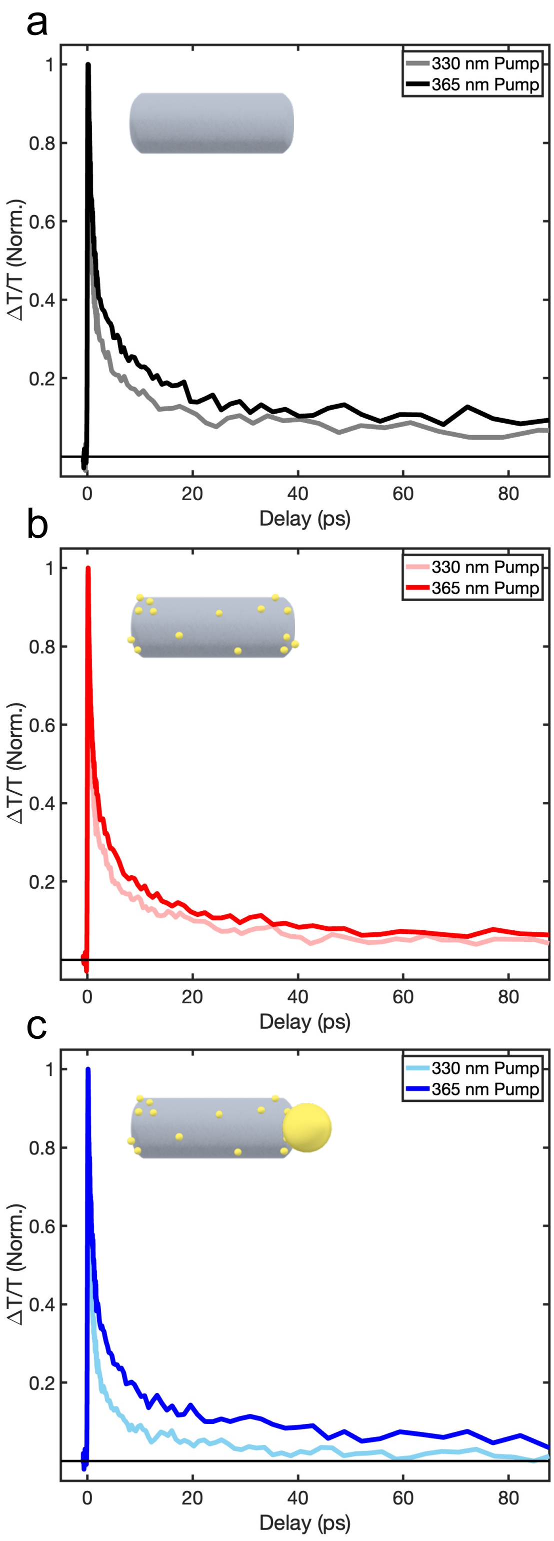

Figure S13. Normalized TA dynamics of the bleach recovery at 380 nm for a) ZnSe NRs and ZnSe-Au HNPs in the b) SAC and c) metal-tip regime, corresponding to the samples shown in Figure 4. Black, red, and blue lines represent near-band-gap excitation at 365 nm (3.40 eV), corresponding to the excitation energy used for the photocatalytic measurements. Grey, pink, and cyan lines represent excess-energy excitation at 330 nm (3.76 eV). Whereas pristine ZnSe (black/grey) and SAC-HNPs (red/pink) show only minor changes upon increasing pump energy, the Au-tipped ZnSe (blue/cyan) reach faster and complete bleach recovery, indicating a more efficient hot electron transfer to the Au tips.

(1) Ravel, B.; Newville, M. ATHENA, ARTEMIS, HEPHAESTUS: Data Analysis for X-Ray Absorption Spectroscopy Using IFEFFIT. *J. Synchrotron Radiat.* **2005**, *12*, 537–541.

(2) Frenkel, A. Solving the 3D Structure of Metal Nanoparticles. *Z. Kristallogr.* **2007**, *222*, 605–611.

(3) Frenkel, A. I.; Hills, C. W.; Nuzzo, R. G. A View from the inside: Complexity in the Atomic Scale Ordering of Supported Metal Nanoparticles. *J. Phys. Chem. B* **2001**, *105*, 12689–12703.

(4) Momma, K.; Izumi, F. VESTA 3 for Three-Dimensional Visualization of Crystal, Volumetric and Morphology Data. *J. Appl. Crystallogr.* **2011**, *44*, 1272–1276.
